# A nomogram model for predicting maternal cardiovascular complications and neonatal adverse outcomes in pregnant patients with pulmonary arterial hypertension

**DOI:** 10.1080/07853890.2025.2541093

**Published:** 2025-08-04

**Authors:** Ruilin Ma, Jianjian Cui, Yanfang Zheng, Hui Tao, Wencong He, Zejun Yang, Yanan Li, Yin Zhao

**Affiliations:** a Department of Obstetrics and Gynecology, Union Hospital, Tongji Medical College, Huazhong University of Science and Technology, Wuhan, China; b The First Affiliated Hospital of Zhengzhou University, Zhengzhou, China; c Shenzhen Huazhong University of Science and Technology Research Institute, Shenzhen China

**Keywords:** Pregnancy, pulmonary arterial hypertension, maternal and neonatal outcomes, prediction model

## Abstract

**Background:**

Pulmonary arterial hypertension (PAH) during pregnancy significantly increases maternal and fetal mortality risk. We developed nomogram prediction models from retrospective data to assess maternal cardiovascular risks and neonatal adverse outcomes.

**Methods:**

Our study included 170 pregnant women, divided into training (70%) and validation (30%) sets. Predictors of outcomes were identified using logistic regression in the training set, and nomograms were constructed to predict maternal cardiovascular complications and neonatal adverse outcomes. Model performance was evaluated through internal validation.

**Results:**

Predictors of cardiovascular complications included severe PAH (OR = 4.80), New York Heart Association (NYHA) classification ≥ III (OR = 25.94), ST-T changes (OR = 25.18), total bilirubin (OR = 1.49), albumin (OR = 0.87) and lactate dehydrogenase level (OR = 1.01). The nomogram showed high predictive accuracy with concordance indices of 0.96 and 0.91, areas under the ROC curve of 0.96 and 0.93. Neonatal outcome predictors included gestational age at termination (OR: 0.93), maternal platelet count level (OR: 0.99), and B-type natriuretic peptide level (OR: 1.00). The corresponding nomogram showed concordance indices in the training set and validation set were 0.92 and 0.73, respectively, with area under the ROC curve values of 0.92 and 0.73.

**Conclusions:**

Nomogram models based on the above factors useful tools for predicting cardiovascular complications and neonatal adverse outcomes in pregnant women with PAH, potentially aiding in early detection and timely intervention. Further validation is needed to confirm their accuracy in broader clinical settings.

## Introduction

Pulmonary arterial hypertension (PAH) during pregnancy represents a serious comorbidity characterized by pathophysiological disorders that elevate pulmonary circulation pressure. The condition manifests primarily through increased pulmonary artery pressure (PAP) and pulmonary vascular resistance (PVR) [1–3]. The mean pulmonary artery pressure (mPAP) is 14 ± 3.3 mmHg at resting state, with a normal upper limit of 20.6 mmHg [2,4]. In the past, PAH was defined hemodynamically by mPAP ≥ 25 mmHg as measured by right heart catheterization (RHC) at rest [1]. However, in 2022, the European Society of Cardiology (ESC) and the European Respiratory Society (ERS) revised this definition, lowering the cutoff to 20 mmHg [5]. The prevalence of PAH ranges from 15 to 60 cases per million, with a higher incidence in women than men, accounting for 60% to 80% of cases, predominantly among those of reproductive age [1,6,7].

Maternal hemodynamic changes during pregnancy, including increased blood volume and cardiac output as well as reduced systemic vascular resistance due to progesterone and nitric oxide, lead to increased oxygen consumption and a hypercoagulable state [3,8]. Consequently, pregnant patients with PAH experience impaired pulmonary vasodilation and poor tolerance to these physiological changes. Such patients are at heightened risk for severe complications, including refractory right heart failure (RHF), pulmonary hypertensive crisis (PHC), pulmonary artery embolism, venous thrombosis, and even death [1]. Therefore, PAH during pregnancy remains a high-risk condition with reported maternal mortality rates ranging from 25% to 56% [9,10], primarily due to heart failure (HF). The World Health Organization (WHO) categorizes PAH as class IV heart disease and pregnancy is not recommended for such patients [11]. According to the 2018 ESC Guidelines for the management of cardiovascular disease during pregnancy [2], it is recommended that PAH patients, particularly those at class IV risk, receive comprehensive management from a multidisciplinary team, including at least cardiologists, obstetricians and anesthesiologists, to determine the delivery plan and postpartum care plan.

Despite these recommendations, many women with PAH still choose pregnancy or continue with an unplanned pregnancy. In addition, some patients with PAH only have non-specific symptoms such as dyspnea after fatigue in the early stage of PAH onset, and the diagnosis of PAH is usually delayed as PAH coincides with the symptoms of early pregnancy, and a definite diagnosis of PAH cannot be made for the first time until the middle and late stages of pregnancy [12]. As the pregnancy progresses, the additional strain on maternal circulation and resultant fetal hypoxia may cause severe complications, including fetal growth restriction, premature delivery, fetal distress, and even neonatal death [13].

Therefore, an early and effective assessment of pregnancy risk for these patients is crucial to improve their prognosis. At present, several risk classification systems are utilized, including the Modified World Health Organization Classification of Maternal Cardiovascular Risk (mWHO) [14], the Cardiac Disease in Pregnancy Study (CARPREG) risk score [15], and the Zwangerschap-bij-Aangeboren-HARtAfwijkingen (ZAHARA) [16]. The current risk prediction systems provide pregnancy risk classification warnings for women of childbearing age with various cardiovascular diseases, such as PAH and congenital heart disease (CHD). However, there is a scarcity of research focused on identifying predictors and developing prediction models for PAH during pregnancy. Developing a risk assessment and early warning model that aligns with contemporary medical advancements is critical. Such a model would enable timely and optimal consultations, ensuring favorable pregnancy outcomes for patients with PAH, and facilitating health education for those advised against continuing their pregnancy. This would contribute to timely pregnancy termination when necessary, aiding in clinical diagnosis and treatment, and reducing maternal and infant complications. The recent shifts in China’s family planning policies, transitioning from a one-child policy to two-child and three-child policy, have increased the number of elderly and high-risk pregnant women, and there may be changes in the predictors. The previous prediction systems may not be able to meet the current situation and need to be constantly improved to increase the sensitivity and specificity of the prediction tools. This study aimed to analyze the factors influencing cardiovascular complications and neonatal adverse outcomes in pregnant patients with PAH and to develop nomogram models for rapid clinical assessment of the patient’s condition.

## Research design and methods

### Participants

This study retrospectively analyzed 170 cases of pulmonary arterial hypertension (PAH) in pregnancy, after excluding 21 cases with unavailable data and 18 cases with incomplete data, managed at the Department of Obstetrics of Union Hospital, Tongji Medical College, Huazhong University of Science and Technology, between January 1, 2010, and February 1, 2022.

Inclusion criteria: Patients were included if they met all of the following requirements: (1) pregnant women diagnosed with PAH; (2) singleton pregnancy; (3) patients with complete case data that were accessible;

Exclusion criteria: Patients were excluded if they met one or more of the following conditions: (1) woman without PAH; (2) multiple pregnancy; (3) patients with incomplete case data or the data were unavailable; (4) pregnancies complicated by non-cardiovascular internal medical or surgical conditions (e.g. appendicitis, hepatitis).

Diagnostic criteria of PAH: According to the 2015 ESC/ERS guidelines for the diagnosis and treatment of PAH [7], the diagnostic criteria were as mPAP ≥ 25 mmHg measured by RHC at sea level and resting state. Strict diagnostic criteria should be based on the hemodynamic data of pulmonary circulation obtained by RHC. However, RHC is an invasive examination with certain risks, and wide application of RHC is limited. Pregnant women belong to a special group, and conventional RHC cannot be used to obtain pulmonary artery pressure. Therefore, pulmonary artery systolic pressure (sPAP) is often estimated indirectly through the measurement of peak tricuspid regurgitation velocity using non-invasive cardiac Doppler echocardiography. When sPAP is ≥ 36 mmHg, PAH is diagnosed. This diagnosis is typically made through a collaborative effort between cardiologists and ultrasonographers and is widely used for non-invasive PAH assessment [17].

Classification of PAH: According to the Chinese Expert Consensus on Diagnosis and Treatment of Pregnancy Complicated with Heart Disease (2016) [18], PAH is divided into mild (< 50 mmHg), moderate (50 to 80 mmHg) and severe (≥ 80 mmHg) based on the pulmonary artery pressure.

### Study design of the model for predicting cardiovascular complications

This study included 170 cases, categorized based on the occurrence of cardiovascular complications. The case group had 25 cases of cardiovascular complications and the non-occurrence group had 145 cases. Cardiovascular complications were defined as the presence of any of the following cases (during pregnancy and up to 42 days postpartum): heart failure, thromboembolic events, infective endocarditis, malignant arrhythmia, pulmonary hypertensive crisis, need for perinatal invasive cardiovascular surgery, cardiac death, and aortic dissection [19].

Candidate predictors were selected based on our clinical expertise and preliminary analysis of the collected patient data, aiming to include variables that are clinically relevant and readily measurable in routine obstetric care for pregnant women with pulmonary arterial hypertension. The selected variables encompassed demographic characteristics, clinical symptoms, laboratory test results, and echocardiographic findings, all of which have potential implications for predicting maternal cardiovascular complications. These candidate predictors were then subjected to statistical screening to identify significant factors for inclusion in the predictive model.

The key clinical factors in our study were derived from patient medical histories, nursing records, laboratory biochemical test results, and echocardiography findings. This study collected comprehensive data including general information (age, gestational age at delivery, gravidity, parity, height, admission weight, PAH classification, etiology of PAH, and history of prior heart disease), pregnancy-related comorbidities and complications, and cardiac-related test results such as New York Heart Association (NYHA) classification post-admission, oxyhemoglobin saturation (SpO_2_), and the most recent echocardiogram, electrocardiogram (ECG), and blood biochemistry results before delivery. These biochemistry results encompassed total bilirubin (TBIL), albumin (ALB), complete blood count parameters, platelet count (PLT), coagulation profiles including thrombin time (TT), plasma fibrinogen (FIB), activated partial thromboplastin time (APTT), international normalized ratio (INR), prothrombin time (PT), D-dimer (D-D), B-type natriuretic peptide (BNP), creatine kinase (CK), and lactate dehydrogenase (LDH). In addition, pregnancy outcomes were also included, such as methods of terminating pregnancy, whether admitted to intensive care unit, maternal mortality, postpartum hemorrhage, anesthesia type, fetal distress, Apgar score, whether transferred to Neonatal Intensive Care Unit, birth weight, and neonatal complications. Statistical analyses included difference comparisons between the case group and the control group, and a univariate logistic regression analysis was performed. In addition to statistical significance, clinical relevance and prior knowledge were also considered when determining which variables to retain in the final model. The indicators with differences between groups and possible predictive value were further analyzed using multivariate logistic regression.

The medical records were randomly allocated into the training set and the validation set at the ratio of 7:3. In the training set, initially screened indicators were analyzed to identify independent risk factors. These factors were then assessed using stepwise regression to determine the final predictors. Based on these predictors, a nomogram of the risk prediction model was developed.

In both the training set and validation set, discrimination was evaluated by the concordance index (C-index) obtained *via* internal bootstrap validation to assess stability, and by the area under the ROC curve (AUC). To assess calibration, we performed the Hosmer–Lemeshow goodness-of-fit test by dividing predicted probabilities into ten groups (χ^2^ statistic; *p* > 0.05 indicates good fit) and plotted calibration curves to compare predicted versus observed risks. Finally, decision curve analysis (DCA) was conducted to evaluate the nomogram’s clinical utility.

### Study design of the model for predicting neonatal adverse outcomes

After excluding 36 cases with iatrogenic abortion and induced labor from initial 170, 134 cases remained for the neonatal adverse outcomes prediction model. This model comprised 51 cases in the case group and 83 in the non-occurrence group. Neonatal adverse outcomes were defined as any of the following: preterm birth, neonatal asphyxia, fetal growth restriction, low birth weight, fetal death, or stillbirth.

The study design, including variable selection and modeling approach, was consistent with for the cardiovascular complications prediction model.

### Statistical analysis

Data were entered using Epidata 3.1, analyzed statistically with SPSS Statistics 25, and risk prediction models along with internal validation were constructed using R Studio. The Shapiro-Wilk test, histograms, and Percent-Percent Plots assessed the normality of the data. Normally distributed data were presented as mean ± standard deviation (SD), while non-normally distributed data were shown as median and interquartile ranges [M (P25, P75)]. Categorical data were expressed as frequencies and percentages (%). For data comparison between groups, the independent sample t-test was used for normally distributed numerical data, and the Mann-Whitney test was applied to non-normally distributed numerical data. The Chi-square test was employed for unordered categorical data, and the Mantel-Haenszel Chi-square test for ordered categorical data, with a *p* value of <0.05 considered statistically significant. Fisher’s exact test was utilized when expected frequencies were below five. Univariate logistic regression initially screened risk factors, which were then further analyzed using multivariate logistic regression to calculate odds ratios (OR) and 95% confidence intervals (95% CI), where OR > 1 indicates a risk factor, OR < 1 a protective factor, and OR = 1 no association with the disease. Stepwise regression analysis was used to select predictors, establish the optimal regression equation, and finalize the risk prediction model. Variables with borderline or non-significant statistical results were retained in the final models due to their established clinical relevance and potential impact on clinical decision-making.

## Results

### Baseline characteristics of pregnant patients with PAH

**Figure 1.**
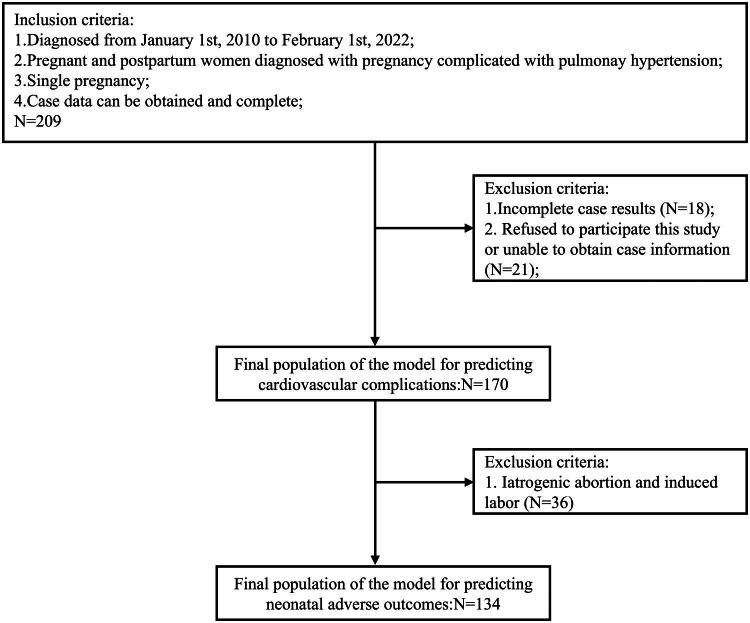
The flow chart of eligibility, inclusion, and exclusion criteria for the study population.

A total of 209 eligible cases were initially identified, and after excluding 21 cases due to unavailable data and 18 cases due to incomplete data, 170 cases were included in the study (Figure 1). The patients’ ages ranged from 18 to 42 years, with a mean age of 28.57 ± 4.75 years. The mean gestational age at termination was 215.46 ± 75.67 days. The mean weight of the participants was 61.23 ± 11.20 kg, and the mean height was 160.16 ± 4.42 cm. Of the 170 patients, 104 were primiparas (61.18%) and 66 were multiparas (38.82%). The New York Heart Association (NYHA) classification distribution was as follows: 8 cases (4.71%) were classified as NYHA I, 103 cases (60.59%) as NYHA II, 53 cases (31.18%) as NYHA III, and 6 cases (3.53%) as NYHA IV. Pregnancy outcomes included 29 iatrogenic abortions in the first trimester (17.06%), 7 induced labors in the second trimester (4.12%), 3 vaginal deliveries (1.77%), 9 cesarean sections to remove the fetus (5.29%), and 122 cesarean deliveries (71.76%), as detailed in Table 1.

**Table 1.** Basic information of 170 pregnant patients with pulmonary arterial hypertension.

	Mean ± SD	Number of cases	Percentage
Age (years)	28.57 ± 4.75		
Gestational age at termination (days)	215.46 ± 75.67		
Weight (Kg)	61.23 ± 11.20		
Height (cm)	160.16 ± 4.42		
Gravidity and parity			
		
-Nulliparity		104	61.18%
-Multiparity		66	38.82%
NYHA classification			
-Class I		8	4.71%
-Class II		103	60.59%
-Class III		53	31.18%
-Class IV		6	3.53%
Methods of terminating pregnancy			
-Iatrogenic abortion in the first trimester		29	17.06%
-Induced labor in the second trimester		7	4.12%
-Vaginal delivery		3	1.77%
-Cesarean section for fetal retrieval		9	5.29%
-Cesarean delivery		122	71.76%
Cause of PAH			
Congenital heart disease		119	70.00%
-Coronary sinus defect		1	
-Atrial septal defect		63	
-Ventricular septal defect		32	
-Complex congenital heart disease		14	
-Patent ductus arteriosus		4	
-Patent foramen ovale		2	
-Papillary muscle dysplasia		1	
-Noncompaction of the ventricular myocardium		2	
Connective tissue disease		28	16.47%
-Rheumatic heart disease		21	
-Systemic lupus erythematosus		6	
-Other types of connective tissue diseases		1	
Valvular heart disease		10	5.88%
Unexplained or idiopathic PAH		7	4.12%
-Unexplained PAH		5	
-Idiopathic PAH		2	
Complications during pregnancy		6	3.53%
-Severe preeclampsia		3	
-Peripartum cardiomyopathy		3	
Detection of PAH			
-Pre-pregnancy		76	44.71%
-After pregnancy		94	55.29%
Presence of cardiac structural changes			
-None		53	31.18%
-Present		117	68.82%
History of treatment for heart disease			
-None		131	77.06%
-Present		39	22.94%
History of cardiac-related surgery before pregnancy			
-None		132	77.65%
-Present		38	22.35%

PAH, pulmonary arterial hypertension.

In a cohort of 170 pregnant patients with PAH, 119 cases (70.00%) were associated with congenital heart disease (CHD). This included one case of coronary sinus defect, 63 cases of atrial septal defect, 32 cases of ventricular septal defect, 14 cases of complex CHD, 4 cases of patent ductus arteriosus, 2 cases of patent foramen ovale, one case of papillary muscle dysplasia, and 2 cases of noncompaction of the ventricular myocardium. Additionally, there were 28 cases (16.47%) of connective tissue disease, including 21 cases of rheumatic heart disease, 6 cases of systemic lupus erythematosus, and 1 case of other connective tissue diseases. The study also found 10 cases (5.88%) of valvular heart disease, 7 cases (4.12%) of unexplained or idiopathic PAH (5 cases of unexplained PAH and 2 cases of idiopathic PAH), and 6 cases (3.53%) of complications during pregnancy (3 cases of severe preeclampsia and 3 cases of peripartum cardiomyopathy), as shown in Table 1.

Among the patients, 44.71% (*n* = 76) were diagnosed with PAH before pregnancy, while 55.29% (*n* = 94) were diagnosed after pregnancy. Cardiac structural changes were present in 68.824% (*n* = 117) of the participants, whereas 31.18% (*n* = 53) had no structural abnormalities. A history of treatment for heart disease was reported by 22.94% (*n* = 39) of patients, while 77.06% (*n* = 131) had no such history. Additionally, 22.35% (*n* = 38) of the patients had undergone cardiac-related surgery prior to pregnancy, with 77.65% (*n* = 132) having no history of surgery, as shown in Table 1.

### Influencing factors of cardiovascular complications in pregnant patients with PAH

Toidentify the predictors of cardiovascular complications, the indicators with difference between the group with cardiovascular complications and the non-occurrence group (Tables S1–S10 in Supplementary Material) and the possible influencing factors were analyzed using univariate logistic regression in the training set (Table 2). These included PAH classification, NYHA classification ≥ III, oxyhemoglobin saturation (SpO_2_), total bilirubin (TBIL), B-type natriuretic peptide (BNP), lactate dehydrogenase (LDH), ICU admissions, fetal distress, ST-T changes on ECG, left atrial hypertrophy, left atrial diameter, and the degree of aortic regurgitation on echocardiogram. Subsequently, these variables were subjected to multivariate logistic regression (Table 3). Notable findings included moderate to severe PAH (OR = 4.80, 95% CI: 1.48–81.06, *p* = 0.03), NYHA ≥ III (OR = 25.94, 95% CI: 2.40–7333.11, *p* = 0.02), ST-T changes (OR = 25.18, 95% CI: 2.64–460.21), TBIL (OR = 1.49, 95% CI: 1.14–2.16, *p* = 0.01), LDH (OR = 1.01, 95% CI: 1.00–1.02, *p* = 0.01), all indicating significant statistical differences as independent risk factors for cardiovascular complications. Conversely, albumin (ALB) (OR = 0.87, 95% CI: 0.64–0.91, *p* = 0.004) was identified as a protective factor.

**Table 2.** Univariate logistic regression analysis of factors influencing maternal cardiovascular complications in pregnant patients with PAH in the training set.

	OR	95% CI	*p* Value
PAH classification			
-Mild	Reference		
-Moderate	0.32	0.02–2.15	0.31
-Severe	3.15	1.05–10.74	0.05
NYHA classification ≥ III	9.46	3.09–35.72	<0.001
Methods of terminating pregnancy			
-Iatrogenic abortion in the first trimester	Reference		
-Induced labor in the second trimester	5.67	0.19–174.46	0.26
-Vaginal delivery	8.50	0.27–289.76	0.18
-Cesarean section to remove the fetus	5.67	0.46–135.87	0.19
-Cesarean delivery	3.07	0.55–57.64	0.30
Gestational age at termination (days)	1.00	0.99–1.01	0.74
Detection of PAH			
-Pre-pregnancy	Reference		
-After pregnancy	2.75	0.91–10.23	0.09
Presence of cardiac structural changes			
-None	Reference		
-Present	0.71	0.25–2.08	0.51
History of treatment for heart disease			
-None	Reference		
-Present	0.47	0.07–1.83	0.34
History of cardiac-related surgery before pregnancy			
-None	Reference		
-Present	1.67	0.65–4.23	0.28
ECG results			
-ST-T changes	7.32	2.20–24.60	0.001
-Left ventricular high voltage	9.800	1.51–79.23	0.02
-Right ventricular high voltage	6.69	1.11–37.90	0.03
-Left atrial hypertrophy	5.73	1.78–18.27	0.003
-Right ventricular hypertrophy	2.18	0.63–6.770	0.19
-Ventricular premature beats	0.92	0.05–5.88	0.94
-Incomplete right bundle branch block	2.53	0.80–7.80	0.08
-Complete the right bundle branch block	1.12	0.056–7.518	0.92
SpO_2_ (%)	0.80	0.653–0.928	0.01
TBIL (umol/L)	1.20	1.084–1.356	0.001
ALB (g/L)	0.80	0.697–0.909	<0.001
Hb (g/L)	1.00	0.982–1.02	0.94
D-D (mg/L)	1.13	1.007–1.452	0.31
BNP (pg/ml)	1.00	1.001–1.003	0.001
LDH (U/L)	1.01	1.000–1.011	0.04
Echocardiogram results			
-Left atrium	1.67	1.003–2.817	0.05
-Interventricular septum thickness	0.510	0.019–16.258	0.69
-Left ventricle	1.79	1.069–3.154	0.03
-A-wave of the mitral valve spectrum	1.75	1.110–2.739	0.01
-Pulmonary valve	0.20	0.031–0.886	0.06
Degree of aortic regurgitation			
-None	Reference		
-A small amount	2.36	0.112–20.058	0.47
-Medium	2.36	0.112–20.058	0.47
-A large amount	10.62	1.619–86.623	0.01
Anesthesia methods			
-No anesthesia is required	Reference		
-Intraspinal anesthesia	0.94	0.276–3.752	0.93
-General anesthesia	1.31	0.307–5.888	0.72
ICU admission			
-No	Reference		
-Yes	10.50	3.416–39.78	<0.001
Fetal distress			
-None	Reference		
-Have	7.31	1.815–9.82	0.004

NYHA, New York Heart Association; PAH, pulmonary arterial hypertension; ECG, electrocardiogram; SpO_2_, oxyhemoglobin saturation; TBIL, total bilirubin; ALB, albumin; Hb, hemoglobin; D-D, D-dimer; BNP, B-type natriuretic peptide; LDH, lactate dehydrogenase.

**Table 3.** Multivariate logistic regression analysis of factors influencing maternal cardiovascular complications in pregnant patients with PAH in the training set.

	OR	95% CI	*p* Value
PAH classification			
-Mild	Reference		
-Moderate	0.22	0.004–7.11	0.42
-Severe	4.80	1.48–81.06	0.03
NYHA classification ≥ III	25.94	2.40–7333.11	0.02
ECG results			
-ST-T changes	25.18	2.64–460.21	0.01
-Left atrial hypertrophy	216.39	0.22–807012.65	0.22
SpO_2_ (%)	1.26	0.91–1.79	0.15
TBIL (umol/L)	1.49	1.14–2.16	0.01
ALB (g/L)	0.87	0.64–0.91	0.004
BNP ≥ 300 pg/ml	1.02	0.07–13.60	0.99
LDH (U/L)	1.01	1.00–1.02	0.01
Echocardiogram results			
-Left atrium	1.57	0.45–7.35	0.51
Degree of aortic regurgitation			
-None	Reference		
-A small amount	0.001	0.00–0.03	0.95
-Medium	0.82	0.01–55.44	0.93
-A large amount	68.09	0.36–106611.53	0.20
ICU admission or not			
-No	Reference		
-Yes	1.72	0.19–17.17	0.63
Fetal distress			
-None	Reference		
-Have	7.45	0.44–121.94	0.14

NYHA, New York Heart Association; ECG, electrocardiogram; SpO_2_, oxyhemoglobin saturation; TBIL, total bilirubin; ALB, albumin; BNP, B-type natriuretic peptide; LDH, lactate dehydrogenase.

### Development and validation of nomogram for predicting cardiovascular complications in pregnant patients with PAH

**Figure 2.**
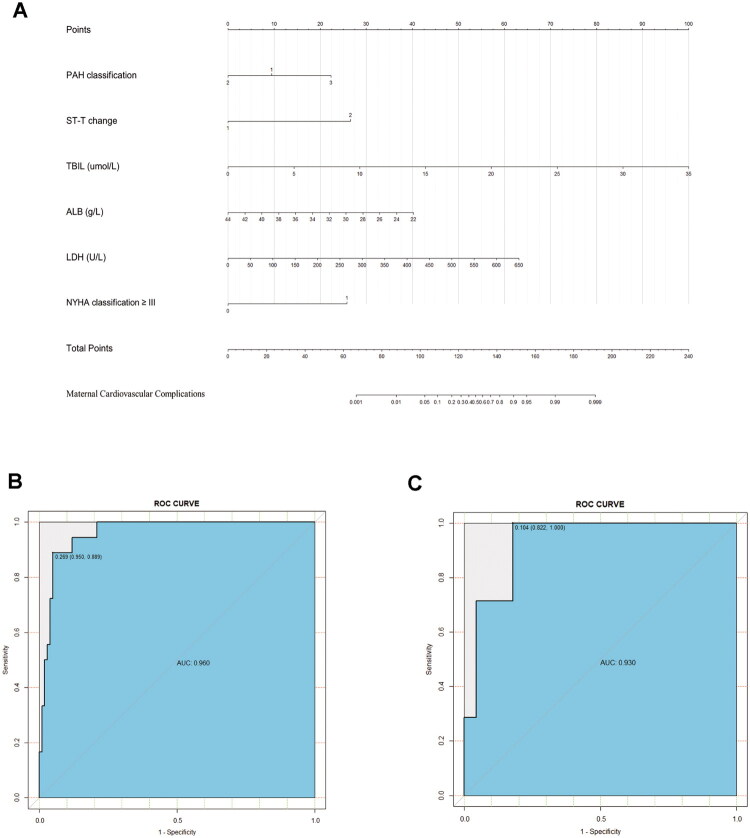
Nomogram for preoperative estimation of cardiovascular complications risk in pregnant patients with pulmonary arterial hypertension (PAH) and its ROC curves in the training set and validation set. (**A**) Nomogram to estimate the risk of cardiovascular complications. To utilize the nomogram, locate each variable on its respective axis, then draw a upwrd line to the points” axis to determine the individual score. Sum all variables scores and project the total tothe“total points” axis to estimate the probabilities of cardiovascular complications, shown at the bottom of the nomogram. For PAH classification, 0 = mild severity, 1= moderate severity, 2 = severe severity. For ST-T changes, 1 = no changes, 2 = presence of changes. For NYHA classification ≥ III, 0 = absence, 1 = presence. **(B)** ROC curve of the nomogram prediction model for cardiovascular complications in the training set (*n* = 118). **(C)** ROC curve of the nomogram prediction model for cardiovascular complications in the validation set (*n* = 52). PAH, pulmonary arterial hypertension. TBIL, total bilirubin; ALB, albumin; LDH, lactate dehydrogenase; NYHA, New York Heart Association; ROC, receiver operating characteristic.

The preliminary predictors for cardiovascular complications-PAH classification, ST-T changes, TBIL, ALB, LDH, and NYHA classification ≥ III were analyzed using forward stepwise logistic regression. Significant predictors were identified and incorporated into a regression model, culminating in the establishment of a nomogram prediction model for cardiovascular complications (Figure 2A). The model showed strong discriminative ability, with AUCs of 0.96 (training set) and 0.93 (validation set), and C-indexes of 0.96 and 0.91, respectively. Although AUC and C-index are theoretically equivalent in logistic regression, we report both as they were calculated using different methods: ROC-based analysis for AUC and bootstrap validation for C-index (Figure 2B,C, Table 4). Although some predictors included in the multivariate regression model were not statistically significant, they were retained due to their established clinical relevance and potential impact on maternal cardiovascular outcomes. This approach ensures that the nomogram model maintains practical clinical applicability despite borderline or non-significant statistical findings.

**Table 4.** Comparison of ROC results and C-index between the training set and the validation set for the nomogram of cardiovascular complications.

	Area under ROC curve	95%CI	Sensitivity	Specificity	Accuracy	Youden index	C-index
Training set	0.96	0.96–0.99	0.89	0.95	0.94	0.84	0.96
Validation set	0.93	0.87–1.00	1.00	0.82	0.85	0.82	0.91

ROC, Receiver operating characteristic.

**Figure 3.**
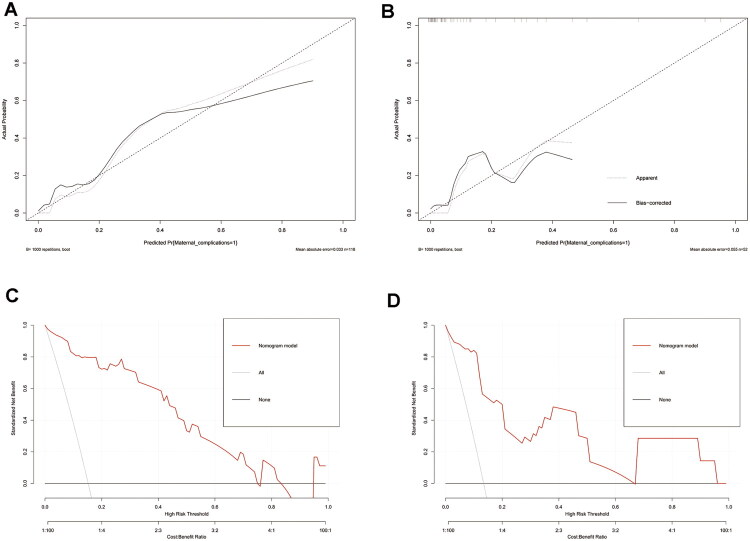
Calibration curves and decision curve analysis (DCA) curves of the nomogram model for preoperative estimation of cardiovascular complications in the training set and validation set (**A**), calibration curve of the nomogram model in the training set. (**B)** Calibration curve of the nomogram model in the validation set. (**C)** DCA curves of the nomogram model in the training set. (**D)** DCA curves of the nomogram model in the validation set. The calibration plots show close agreement between predicted and observed event rates. The Hosmer–Lemeshow goodness-of-fit test yielded *p* = 0.86 (training) and *p* = 0.99 (validation), indicating good model calibration. DCA demonstrates the clinical utility of the model across a range of threshold probabilities.

The Hosmer–Lemeshow goodness-of-fit test yielded *p* values of 0.86 and 0.99 for the training and validation sets, respectively, indicating good model fit. The calibration curves of both sets demonstrated high concordance with the standard curve, indicating excellent model calibration (Figure 3A,B). Decision curve analysis confirmed that the nomogram provided net clinical benefit across a wide range of threshold probabilities (Figure 3C,D).

### Influencing factors of neonatal adverse outcomes in pregnant patients with PAH

To identify predictors of neonatal adverse outcomes in pregnant patients with PAH, the analysis focused on indicators that showed differences between the group experiencing these outcomes and the control group. These indicators, detailed in Tables S11–S20 in Supplementary Material. These indicators underwent univariate logistic regression in the training set (results displayed in Table 5), revealing no statistically significant differences in body weight, PAH classification, ECG findings (including ST-T changes, right axis deviation, left atrial hypertrophy), SpO_2_, TBIL, PLT, LDH, echocardiogram findings (pulmonary valve velocity, degree of tricuspid regurgitation), and ICU admission. However, NYHA classification ≥ III (*p* = 0.03), gestational age at termination of pregnancy (*p* < 0.001), ALB (*p* = 0.006), and BNP (*p* < 0.001) did show statistical significance.

**Table 5.** Univariate logistic regression analysis of predictors for neonatal adverse outcomes in pregnant patients with pulmonary arterial hypertension: Data from the training set.

	OR	95% CI	*p* Value
Maternal weight	0.97	0.93–1.01	0.142
PAH classification			
-Mild	Reference		
-Moderate	1.43	0.49–4.10	0.50
-Severe	2.20	0.80–6.19	0.13
NYHA classification ≥ III	2.69	1.12–6.60	0.03
Gestational age at termination of pregnancy	0.93	0.90–0.96	<0.001
ECG results			
-ST-T changes	1.86	0.48–7.21	0.36
-Right axis deviation	1.59	0.47–5.25	0.44
-Left atrial hypertrophy	2.29	0.70–7.77	0.17
SpO_2_ (%)	0.82	0.65–0.97	0.06
TBIL	1.04	0.976–1.11	0.24
ALB	0.86	0.77–0.96	0.006
PLT	0.99	0.98–1.00	0.06
BNP	1.00	1.00–1.01	<0.001
LDH	1.01	1.00–1.01	0.05
Echocardiogram results			
-Pulmonary valve	1.36	0.62–3.08	0.44
Degree of tricuspid regurgitation			
-None	Reference		
-A small amount	0.87	0.22–3.77	0.84
-Medium	1.50	0.39–6.56	0.57
-A large amount	3.00	0.64–15.94	0.17
ICU admission			
-No	Reference		
-Yes	2.37	1.00–5.71	0.05

NYHA, New York Heart Association; ECG, electrocardiogram; SpO_2_, oxyhemoglobin saturation; TBIL, total bilirubin; ALB, albumin; PLT, platelet count; BNP, B-type natriuretic peptide; LDH, lactate dehydrogenase.

For the prediction model comprising the 134 cases, those indicators demonstrating differences underwent univariate logistic regression in the training set of 93 cases, followed by multivariate logistic regression. The results (Table 6) indicated no significant differences in PAH classification, NYHA classification ≥ III, SpO_2_, ALB, PLT, LDH, and ICU admission. Notably, the gestational age at termination of pregnancy (OR = 0.93, 95% CI: 0.89–0.96, *p* < 0.001) and BNP (OR = 1.00, 95% CI: 1.00–1.01, *p* = 0.005) were significant, suggesting that gestational age at termination acts as a protective factor, while BNP serves as a risk factor for neonatal adverse outcomes in these patients.

**Table 6.** Multivariate logistic regression analysis of predictors for neonatal adverse outcomes in pregnant patients with pulmonary arterial hypertension: Data from the training set.

	OR	95% C	*p* Value
PAH classification			
-Mild	Reference		
-Moderate	2.08	0.50–9.15	0.31
-Severe	0.32	0.04–1.90	0.23
NYHA classification ≥ III	0.92	0.21–3.72	0.91
Gestational age at termination of pregnancy	0.93	0.89–0.96	<0.001
SpO_2_ (%)	1.02	0.84–1.10	0.74
ALB	0.93	0.78–1.09	0.37
PLT	0.99	0.98–1.00	0.12
BNP	1.00	1.00–1.01	0.005
LDH	1.00	0.99–1.01	0.29
ICU admission			
-No	Reference		
-Yes	1.11	0.21–5.23	0.90

PAH, pulmonary arterial hypertension; NYHA, New York Heart Association; SpO2, oxyhemoglobin saturation; ALB, albumin; PLT, platelet count; BNP, B-type natriuretic peptide; LDH, lactate dehydrogenase.

### Development and validation of a nomogram for predicting neonatal adverse outcomes in pregnant patients with PAH

**Figure 4.**
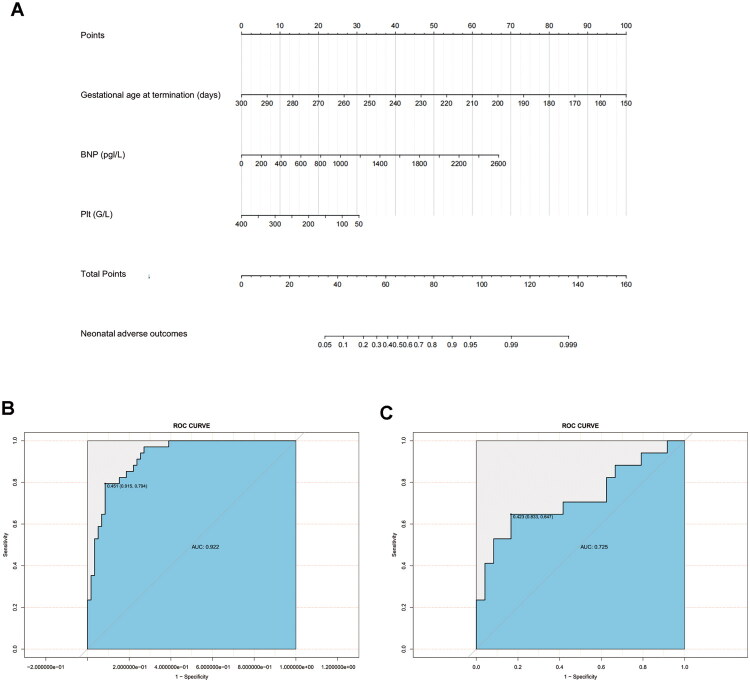
Nomogram for prediction of neonatal adverse outcomes risk in pregnant patients with pulmonary arterial hypertension (PAH) and its ROC curves in the training set and validation set (**A**), nomogram to estimate the risk of neonatal adverse outcomes. (**B)** ROC curve of the nomogram prediction model for neonatal adverse outcome in the training set (*n* = 93). (**C)** ROC curve of the nomogram prediction model for neonatal adverse outcome in the validation set (*n* = 41). BNP, B-type natriuretic peptide; PLT platelet count; ROC, receiver operating characteristic.

Indicators that potentially predict neonatal adverse outcomes, including PAH classification, NYHA classification ≥ III, gestational age at termination of pregnancy, SpO_2_, ALB, PLT, BNP, LDH, and ICU admission were included for stepwise regression analysis. Finally, gestational age at termination of pregnancy, BNP, and PLT were identified as significant predictors. Based on these findings, a regression model was constructed and a nomogram for predicting neonatal adverse outcomes was developed (Figure 4A).

The ROC curves of the prediction model in both the training and validation sets (Figure 4B,C) showed AUC of 0.92 and 0.72, respectively, indicating a reasonable level of discriminative ability. The c-index of the training and validation sets was 0.92 and 0.73, respectively, suggesting the model demonstrates credible performance, though further validation in larger, diverse populations is necessary to confirm its robustness (Table 7). Although the AUC decreased in the validation set, this may be due to limited sample size and data variability. Nevertheless, an AUC of 0.72 is still considered clinically acceptable for early risk prediction.

**Table 7.** Comparison of ROC results and C-index between the training set and the validation set for the nomogram of neonatal adverse outcomes.

	Area under ROC curve	95%CI	Sensitivity	Specificity	Accuracy	Youden index	C-index
Training set	0.92	0.87–0.97	0.79	0.92	0.87	1.71	0.92
Validation set	0.73	0.56–0.90	0.65	0.83	0.76	1.48	0.73

ROC, Receiver operating characteristic.

**Figure 5.**
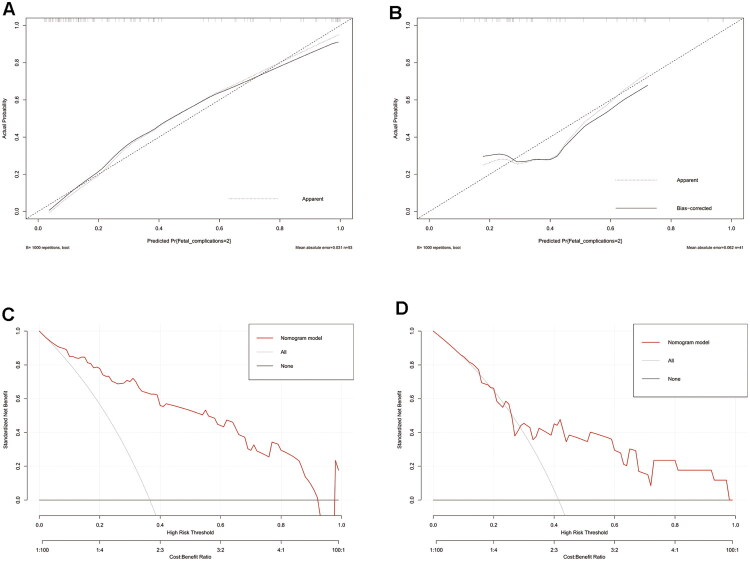
Calibration curves and decision curve analysis (DCA) of the nomogram model for preoperative estimation of neonatal adverse outcomes in the training set and validation set (**A**), calibration curve of the nomogram model in the training set. (**B)** Calibration curve of the nomogram model in the validation set. (**C**) DCA curves of the nomogram model in the training set. (**D)** DCA curves of the nomogram model in the validation set. The calibration plots show close agreement between predicted and observed event rates. The Hosmer–Lemeshow goodness-of-fit test yielded *p* = 0.38 (training) and *p* = 0.81 (validation), indicating adequate model calibration. DCA confirms the clinical utility of the model across a wide range of threshold probabilities.

The Hosmer–Lemeshow goodness-of-fit test yielded *p* values of 0.38 for the training set and 0.81 for the validation set, both exceeding 0.05, which indicates an adequate fit of the model. The calibration curves of the training and validation sets, illustrated in Figure 5A,B, which show that the prediction curve aligns closely with the standard curve, indicating good calibration of the prediction model. Decision curve analysis further confirmed that the nomogram provides net clinical benefit across a wide range of threshold probabilities (Figure 5C,D).

To further explore the potential interaction between maternal cardiovascular complications and neonatal adverse outcomes, we performed an exploratory classification tree analysis. This model stratified patients into subgroups based on the presence or absence of either or both complications, offering additional visual insights into potential overlapping risk profiles. The results of this analysis are presented in Supplementary Figure S1.

## Discussion

### Risk factors for cardiovascular complications and neonatal adverse outcomes in pregnant patients with PAH

PAH in pregnancy is associated with significant cardiovascular complications, including maternal death and heart failure, as well as fetal and neonatal adverse outcomes [20]. The condition’s risk factors warrant further investigation. Our study identified PAH classification, NYHA classification ≥ III, ST-T changes, elevated TBIL and LDH were risk factors for cardiovascular complications in pregnant patients with PAH, while decreased ALB levels acted as a protective factor. Additionally, gestational age at termination of pregnancy was a protective factor for neonatal adverse outcomes, while elevated BNP was a risk factor. Effective early identification, monitoring, and management of pregnant patients with PAH are critical to enhancing pregnancy outcomes and maternal survival. This study provides reference indicators for the clinical screening of patients with a high risk of adverse outcomes and is helpful for early intervention and management of such patients.

The primary cause of mortality in patients with PAH is right heart overload, leading to right heart failure and triggering a cascade of pathological responses [21,22]. BNP serves as a crucial biomarker for diagnosing and predicting heart failure, often regarded as the ‘sentinel’ for heart failure monitoring [23]. Hendriks et al.’s meta-analysis emphasized that BNP monitoring is vital for the risk stratification of PAH patients, with elevated levels strongly linked to increased mortality [24]. Additionally, in the context of pregnancy, studies indicate that BNP is a significant predictor of cardiovascular events in PAH patients associated with congenital heart disease (CHD), with levels ≥ 300 ng/L posing an independent risk [25]. The results of this study are slightly different. This study further explored BNP’s relationship to neonatal adverse outcomes, suggesting that myocardial stress and right ventricular hypertrophy from PAH, coupled with increased pulmonary vascular resistance, lead to significant BNP secretion. This may be due to the myocardial stress and right ventricular hypertrophy caused by PAH, along with the increased pulmonary vascular resistance in the mother, resulting in excessive stretching of myocardial cells that produce a large amount of BNP; consequently the failure of maternal cardiac function leads to fetal blood supply problems, leading to adverse outcomes for the fetus and newborn [26]. Although univariate analysis identified higher BNP levels in the group with cardiovascular complications compared to controls (OR = 1.00, 95% CI: 1.00–1.01, *p* = 0.001), multivariate analysis did not support BNP as a predictor. One potential reason for discrepancies might be that while case data were consistently collected as the last BNP results before delivery, BNP collection in clinical practice often occurs significantly earlier than the time of delivery for some patients. Consequently, BNP levels may fluctuate substantially, failing to accurately represent pre-delivery levels and leading to data deviations. Besides, the small sample size might also affect the findings. Moreover, the literature suggests that the baseline of BNP and the dynamic changes in BNP more accurately reflect the risk in patients with PAH [26]. A prospective study of 1246 patients with congestive heart failure (CHF), demonstrated that variations in BNP levels are more indicative of long-term prognosis in heart failure (HF) patients than absolute BNP levels [27].

PAH classification and maternal function grade are critical for assessing PAH in pregnancy. Many studies consistently show that severe PAH during pregnancy correlates with poorer outcomes compared to mild PAH, with higher incidences of serious adverse events, such as mortality [28–30]. This study identifies PAH classification as an independent risk factor for maternal cardiovascular complications. Specifically, the risk of complications in patients with severe PAH is 4.80 times greater than in those with mild PAH. Additionally, NYHA classification ≥ III was also an independent risk factor, increasing the likelihood of complications by 25.94 times. While the NYHA classification is a subjective measure, it remain widely used and effective for prognostic evaluations of patients with PAH [31,32].

Electrocardiography (ECG) is the most fundamental and routine cardiac assessment tool. Although increasingly utilized in prenatal examinations, ECG still does not receive adequate attention in clinical practice. Studies indicate that ECG, as a cheap and non-invasive examination method, plays an important role in diagnosing PAH [33–35]. Ley L et al. observed specific ECG patterns in pregnant patients with PAH, including *R* ≤ 2 mm in lead I, *S* ≤ 2 mm in V_1_, R/*S* ≥ 1 in V_1_, R/*S* ≤ 1 in V_6_, and the electric axis ≥ 110°, and qR appears in V_1_ [36]. This study also included a statistical analysis of patients’ ECGs, which identified ST-T changes as independent risk factors for cardiovascular complications (OR = 25.18, 95% CI: 2.64–460.21, *p* = 0.01). Typically associated with coronary artery insufficiency, ST-T segment changes are often occur in patients with coronary heart disease, hypertensive heart disease, etc. [37]. This study suggests that ST-T changes, important indicators for predicting cardiovascular complications, may result from continuous and irreversible damage to myocardial cells at the negative pole of ventricular myocytes, a consequence of deteriorating cardiac function as PAH progresses.

Zhang Jun et al. founded that elevated TBIL and WHO class III or IV are independent risk factors for perinatal complications during cesarean section in patients with PAH [38]. This finding aligns with our study, which identifies elevated TBIL as a risk factor for cardiovascular complications included in the predictive model. Additionally, several studies have linked increases in TBIL to reduced survival rates and poor prognosis in patients with PAH [39,40], reflecting the hemodynamic parameters such as the severity of right atrial pressure and tricuspid regurgitation to a certain extent [41]. This may be because right atrial overload and tricuspid regurgitation, caused by PAH, lead to increased hepatic venous pressure, resulting in elevated TBIL [42]. Elevated TBIL has also been poor coronary flow in patients with unstable angina pectoris [43]. While the prognostic significance of TBIL in cardiovascular disease is clinically beneficial, its specific mechanism remains unclear, necessitating further research.

Dai L et al. identified ALB as an important predictor of maternal death in PAH during pregnancy [44], findings that align with those of our study, which also identified ALB as a protective factor for cardiovascular complications (OR = 0.87, 95% CI: 0.64–0.91, *p* = 0.004). Decreased ALB levels are associated with an increased risk of cardiovascular complications. Commonly used to assess malnutrition, ALB has been shown to predict prognosis in patients with heart failure and PAH, and it is an independent risk factor for mortality at the 14th, 28th and 90th days post-CHF diagnosis [45–47]. A prospective clinical study by Kent et al. revealed that low baseline ALB levels were independently associated with reduced 4-year survival in patients with HF and secondary mitral regurgitation [48]. This association may stem from ALB’s role in maintaining colloid osmotic pressure and facilitating ligand binding and nutrient transport. Reductions in ALB may disrupt colloid osmotic balance, increase oxidative stress, and heighten inflammatory responses, thereby precipitating adverse outcomes.

The study by Hu Enci et al. shows that the increased serum lactate dehydrogenase (S-LDH) level is an independent risk factor for the poor prognosis in patients with idiopathic pulmonary arterial hypertension (iPAH) [49]. Similarly, Wang Xuefang et al. have identified that LDH levels can indicate the severity of PAH related to CHD [50]. Our study further corroborates that high LDH levels are an independent risk factor for cardiovascular complications (OR = 1.01, 95% CI: 1.00–1.02, *p* = 0.01). LDH, a cytoplasmic enzyme found in cardiac and skeletal muscles, liver, and other organs, is involved in anaerobic glycolysis. Its levels are indicative of the number of dead or damaged cells [51]. Elevated LDH has been consistently linked to malignant tumors [52], hypertensive heart disease [53], CHD [11], and other diseases. S-LDH levels also correlate significantly with arterial stiffness and the risk of cardiovascular disease in ten years [54]. Elevated LDH levels in patients with PAH may be attributed to impaired liver function caused by prolonged hypoxia, in addition to the important role of LDH-related glycolysis and pulmonary vascular remodeling [55].

In addition to BNP, this study highlights the significant role of platelet count in predicting neonatal adverse outcomes. Decreased PLT and PLT dysfunction are crucial factors in thrombosis, which may lead to an imbalance between coagulation and anticoagulation, abnormal placental microcirculation, increased vascular reactivity, and poor placental function, subsequently resulting in neonatal developmental disorders. PLT has been linked to the severity of several pregnancy-related diseases. Lin SS et al. demonstrated that PLT could predict the severity of eclampsia [56], and similar results were found by Sahbaz A et al. in their research on gestational diabetes mellitus [57].

Further studies indicate that maternal PLT levels in early pregnancy are a vital biological predictor of neonatal birth weight in older pregnant women [58], and have also been utilized as early predictors of neonatal respiratory distress syndrome and neonatal sepsis [59].

### Risk prediction model for adverse outcomes in pregnant patients with PAH

At present, research on risk prediction models for adverse outcomes in pregnant patients with PAH is limited, necessitating further study. Several established pregnancy risk stratification systems such as the modified WHO [14], CARPREG [15], and ZAHARA [16] are available for women with cardiovascular conditions including PAH and CHD. These systems are designed to alert healthcare providers about potential risks during pregnancy. However, specific models focusing on PAH-related pregnancy complications remain scarce.

Chen Shixin et al. developed a model predicting adverse pregnancy outcomes (maternal death, premature delivery, iatrogenic abortion, neonatal asphyxia, or death) in this patient group, incorporating predictors like dyspnea, pulmonary artery pressure, N-terminal pro-B-type natriuretic peptide (NT-proBNP), and anesthesia methods [60].

Similarly, Dai L et al. found NT-pro BNP, pulmonary artery systolic pressure (PASP), and low albumin (ALB) levels to be effective predictors of maternal mortality [44]. Additionally, Chen Yuqin et al. developed nomogram models for predicting maternal death or heart failure (HF) and fetal or neonatal mortality or smallness for gestational age. In their research, type I respiratory failure, NYHA classification, NT-pro BNP levels exceeding 1400 ng/L, arrhythmias, and eclampsia were identified as independent risk factors for maternal death or HF. Furthermore, type I respiratory failure, arrhythmias, cesarean sections under general anesthesia, parity, platelet count (PLT), fibrinogen (FIB), and left ventricular end-systolic diameter emerged as crucial predictors for fetal or neonatal adverse outcomes [61]. These models primarily address maternal and neonatal mortality, with no current research extending these predictions to other serious cardiovascular complications that might affect pregnant and postpartum women. Despite these developments, there remains a gap in models predicting other serious cardiovascular complications during pregnancy.

In this study, two targeted nomogram risk prediction models were developed, one for cardiovascular complications and another for neonatal adverse events in pregnant patients with PAH. These models demonstrated good discrimination, accuracy, and clinical utility upon validation. Both the cardiovascular complication and neonatal outcome prediction models demonstrated high discrimination in the training set (AUCs of 0.96 and 0.92, respectively). In the validation set, the AUCs decreased to 0.93 and 0.72, respectively, but remained at an acceptable level for clinical risk prediction. This research equips clinicians with a straightforward risk prediction tool with utilizes readily accessible data, aiding physicians in the early identification and management of high-risk patients. Additionally, it offers valuable guidance on prognosis and perinatal care, though further validation is needed to fully establish their reliability in diverse clinical settings.

### Pregnancy management plan for patients with PAH

All patients with pulmonary arterial hypertension (PAH), or pregnant women newly diagnosed with PAH, should be immediately referred to a tertiary care center with a well-established cardiology department for treatment and management [11]. The treatment team should include, at a minimum, a cardiologist, obstetrician, anesthesiologist, and neonatologist. An individualized pregnancy management plan should be developed through multidisciplinary consultations [5,62]. Throughout the pregnancy, patients should strictly adhere to the scheduled prenatal check-ups and receive regular monitoring by the multidisciplinary team. In the first and second trimesters, check-ups should be conducted monthly, and in the third trimester, weekly [63].

Reports of successful management of such patients during pregnancy are valuable for learning and reference. Hemnes and colleagues suggest that in the late stages of pregnancy, echocardiography should be performed every 1–2 weeks to monitor cardiac function and adjust medication dosages accordingly [63]. Kiely and colleagues [62] recommend developing a detailed, individualized written management plan for such patients, which should include contingency plans for all possible scenarios. This plan should cover emergency and elective cesarean delivery protocols, choices for anesthesia methods and administration, strategies for managing hypotension and bradycardia, as well as treatment plans for worsening pulmonary arterial hypertension. Coursen and colleagues [18] provide detailed recommendations for multidisciplinary consultations. Their protocol includes an initial six-minute walk test and echocardiographic monitoring every three months or when symptoms change. Multidisciplinary consultations assess both maternal and fetal conditions, as well as the severity of pulmonary arterial hypertension. In mid-pregnancy, the obstetrician evaluates the progression of pregnancy and plans the delivery. In late pregnancy, the focus is on determining the timing and mode of delivery. After admission, a simulated delivery workflow is established, with multidisciplinary consultations taking place 48 h before the planned delivery, on the day of delivery, the day after delivery, and on the day of discharge to address any potential issues that may arise.

## Limitations

This study has several limitations. Firstly, as a single-center retrospective study with a limited sample size, there is potential for recall and selection biases. In particular, the validation dataset was relatively small, which reduces statistical power, increases the risk of model overfitting, and limits the stability and generalizability of the predictive models. Therefore, the results should be interpreted cautiously and considered preliminary. Multicenter studies with larger sample sizes will be needed to validate our findings and minimize these biases. Additionally, while echocardiograms have limitations in assessing the severity of PAH, right heart catheterization (RHC) is not advisable during the perinatal stage due to its invasive nature, resulting in a lack of precise hemodynamic data. Furthermore, the absence of long-term follow-up data also limits our understanding the disease’s progression and long-term outcomes. Incorporating follow-up studies in future research will be critical to addressing this gap. Lastly, although this study conducted an internal validation of the prediction model, there is still room for improvement in its predictive ability. In addition, other potentially influential factors such as socioeconomic status, access to care, and patient preferences were not available in our retrospective dataset and thus could not be included in the present analysis. The lack of these variables may further limit the model’s comprehensiveness and generalizability, and should be addressed in future prospective studies. Given these constraints, future external validation through larger, prospective multicenter studies will be essential to strengthen the robustness and clinical applicability of the predictive models.

## Conclusion

In conclusion, this study identified key risk factors for cardiovascular complications and neonatal adverse outcomes in pregnant patients with PAH and developed two novel nomogram prediction models that demonstrated reasonable discrimination, calibration, and clinical utility. These findings provide valuable insights for the early assessment of pregnant patients with PAH and the creation of personalized perinatal management plans. However, the nomogram models require prospective external validation on a larger scale, and further research is needed before they can be widely implemented in clinical practice.

## Supplementary Material

Supplemental online material.docx

## Data Availability

All data generated or analyzed during this study are included in this published article. Further inquiries can be directed to the corresponding author (Email: zhaoyin@hust.edu.cn).
