# Supplementary material for: A nomogram model for predicting maternal cardiovascular complications and neonatal adverse outcomes in pregnant patients with pulmonary arterial hypertension
Source: Ann Med. 2025 Aug 4;57(1):2541093. doi: 10.1080/07853890.2025.2541093 (PMC12322992; doi:10.1080/07853890.2025.2541093)
Supplement: Supplemental online material.docx [file IANN_A_2541093_SM5189.docx]

**Supplemental online material**

**Figure S1.**Exploratory classification tree analysis stratifying pregnant patients with PAH based on the occurrence of maternal cardiovascular complications and neonatal adverse outcomes.


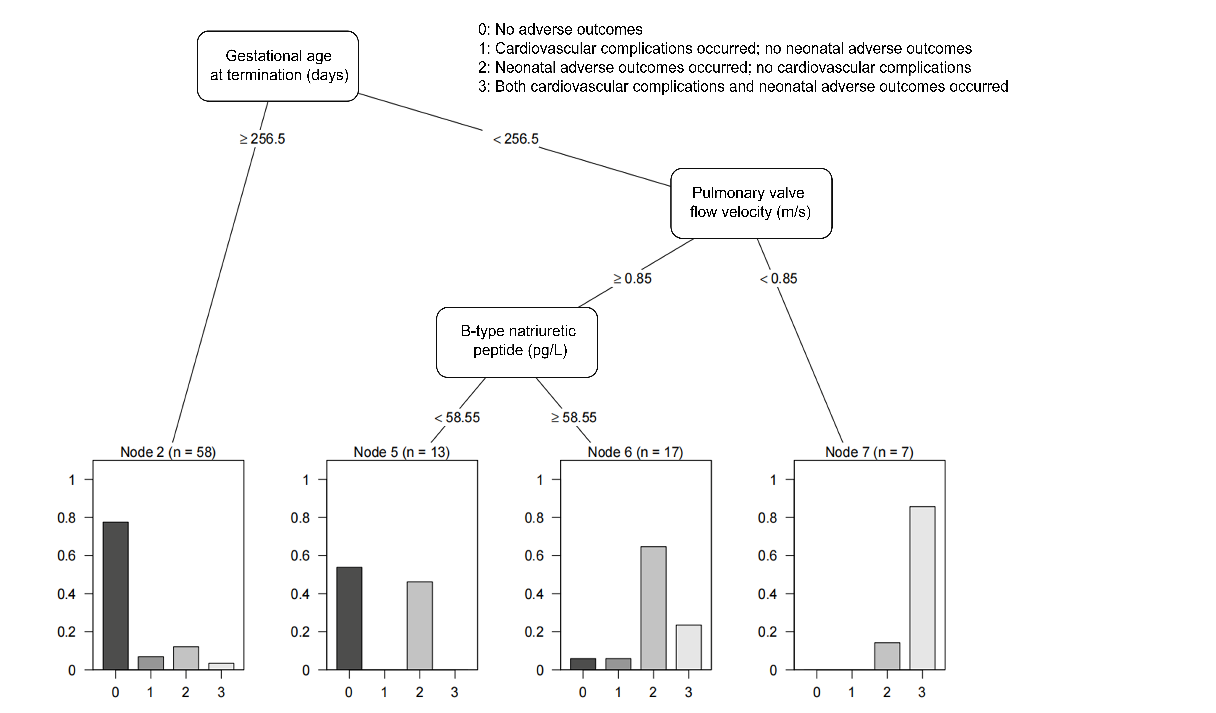


This analysis aimed to provide a preliminary visualization of how patients clustered according to the presence of either or both complications. Patients were categorized into three groups: (1) those with only cardiovascular complications, (2) those with only neonatal adverse outcomes, and (3) those with both. The tree structure offers additional insights into shared and divergent clinical features among these subgroups. This figure is intended to supplement the main analysis and is not part of the primary predictive model.

**Table S1 Comparison of clinical data features between training and validation sets for the prediction model of cardiovascular complications in pregnant patients with pulmonary arterial hypertension.**

|  | | Validation set (n=52) | Training Set (n=118) | P-value |
| --- | --- | --- | --- | --- |
| **Age（years）** | | 29.50 ± 4.82 | 28.10 ± 4.55 | 0.07 |
| **Gravidity** | | 2.40 ± 1.61 | 1.96 ± 1.22 | 0.08 |
| **Parity** | | 0.54 ± 0.64 | 0.42 ± 0.63 | 0.28 |
| **Height (cm)** | 160.00 ± 4.51 | 160.00 ± 4.40 | 0.90 |  |
| **Weight (Kg)** | | 62.20 ± 12.60 | 60.80 ± 10.60 | 0.48 |
| **Gestational age at termination (days)** | | 209.00 ± 87.20 | 219.00 ± 70.20 | 0.47 |
| **Methods of terminating pregnancy ( n, %)** | |  |  | 0.44 |
| -Iatrogenic abortion in the first trimester | 11 (21.15%) | 18 (15.25%) |  |  |
| -Induced labor in the second trimester | 3 (5.77%) | 4 (3.39%) |  |  |
| -Vaginal delivery | 0 (0.00%) | 3 (2.54%) |  |  |
| -Cesarean section to remove the fetus | 1 (1.92%) | 8 (6.78%) |  |  |
| -Cesarean delivery | 37 (71.15%) | 85 (72.03%) |  |  |
| **PAH classification ( n, %)** | |  |  | 0.71 |
| -Mild | | 22 (42.31%) | 47 (39.83%) |  |
| -Moderate | | 9 (17.31%) | 27 (22.88%) |  |
| -Severe | | 21 (40.39%) | 44 (37.29%) |  |
| **Cause of PAH** **( n, %)** | |  |  | 0.13 |
| -Unexplained or idiopathic PAH | | 4 (7.69%) | 3 (2.54%) |  |
| -Connective tissue disease | | 5 (9.62%) | 24 (20.34%) |  |
| -Congenital heart disease | | 36 (69.23%) | 82 (69.49%) |  |
| -Valvular heart disease | | 5 (9.62%) | 5 (4.24%) |  |
| -Complications during pregnancy | | 2 (3.85%) | 4 (3.39%) |  |
| **NYHA classification ( n, %)** | |  |  | 0.28 |
| -Class I | | 2 (3.85%) | 6 (5.08%) |  |
| -Class II | | 32 (61.54%) | 71 (60.17%) |  |
| -Class III | | 14 (26.92%) | 39 (33.05%) |  |
| -Class IV | | 4 (7.69%) | 2 (1.69%) |  |
| **Detection of PAH( n, %)** | |  |  | 0.16 |
| -Pre-pregnancy | | 28 (53.85%) | 48 (40.68%) |  |
| -After pregnancy | | 24 (46.15%) | 70 (59.32%) |  |
| **Presence of Cardiac Structural Changes** **( n, %)** |  |  | 0.79 |  |
| -None | 15 (28.85%) | 38 (32.20%) |  |  |
| -Present | 37 (71.15%) | 80 (67.80%) |  |  |
| **History of treatment for heart disease** **( n, %)** | |  |  | 0.16 |
| -None | | 36 (69.23%) | 95 (80.51%) |  |
| -Present | | 16 (30.77%) | 23 (19.49%) |  |
| **History of cardiac-related surgery before pregnancy ( n, %)** | |  |  | 0.25 |
| -None | | 37 (71.15%) | 95 (80.51%) |  |
| -Present | | 15 (28.85%) | 23 (19.49%) |  |
| **ECG results** **( n, %)** | |  |  |  |
| -Sinus tachycardia | 9 (17.308%) | 19 (16.10%) | 1.00 |  |
| -ST-T changes | 10 (19.23%) | 15 (12.71%) | 0.38 |  |
| -T wave changes | | 5 (9.62%) | 13 (11.02%) | 1.00 |
| -Left ventricular high voltage | 2 (3.85%) | 5 (4.24%) | 1.00 |  |
| -Right ventricular high voltage | | 1 (1.92%) | 6 (5.08%) | 0.68 |
| -Right axis deviation | | 6 (11.54%) | 20 (16.95%) | 0.50 |
| -Left atrial hypertrophy | | 4 (7.69%) | 17 (14.41%) | 0.33 |
| -Right atrial hypertrophy | 1 (1.92%) | 1 (0.85%) | 0.52 |  |
| -Right ventricular hypertrophy | | 8 (15.38%) | 20 (16.95%) | 0.98 |
| -Clockwise rotation | | 3 (5.769%) | 11 (9.32%) | 0.56 |
| -Atrial fibrillation | 0 (0.00%) | 1 (0.85%) | 1.00 |  |
| -Abnormal Q wave | 4 (7.69%) | 3 (2.54%) | 0.20 |  |
| -Ventricular premature beats | 2 (3.85%) | 7 (5.93%) | 0.72 |  |
| -Incomplete right bundle branch block | 11 (21.15%) | 32 (27.12%) | 0.53 |  |
| -Complete right bundle branch block | | 5 (9.62%) | 6 (5.08%) | 0.32 |
| -Paroxysmal supraventricular tachycardia | | 1 (1.92%) | 0 (0.00%) | 0.31 |
| **SpO_2_** (**%)** | | 95.60 ± 13.50 | 97.40 ± 2.88 | 0.36 |
| **TBIL** (**umol/L**) | | 11.60 ±7.60 | 9.66 ± 4.78 | 0.09 |
| **ALB** (**g/L**) | | 31.40 ± 4.98 | 32.30 ± 4.33 | 0.26 |
| **WBC** (**G/L**) | | 8.15 ± 2.67 | 8.59 ± 2.43 | 0.31 |
| **Hb** (**g/L**) | | 112.00 ± 21.80 | 114.00 ± 27.20 | 0.64 |
| **Hct** (**%**) | | 33.90 ± 5.75 | 34.10 ± 6.35 | 0.86 |
| **RBC** (**T/L**) | | 3.81 ± 0.70 | 4.36 ± 4.66 | 0.22 |
| **Plt** (**G/L**) | | 186.00 ± 58.50 | 198.00 ± 59.30 | 0.26 |
| **TT** (**s）** | | 19.70 ± 21.20 | 16.30 ± 2.07 | 0.25 |
| **FIB** (**g/L**) | | 4.52 ± 4.72 | 4.18 ± 1.05 | 0.61 |
| **APTT** (**s**) | | 33.10 ± 7.18 | 34.00 ± 5.96 | 0.45 |
| **INR** | | 1.25 ± 1.67 | 1.12 ± 1.04 | 0.62 |
| **PT** (**s**) | | 12.90 ± 2.63 | 13.10 ± 2.44 | 0.55 |
| **D-D** (**mg/L**) | | 1.57 ± 1.72 | 1.95 ± 6.81 | 0.57 |
| **BNP** (**pg/ml**) | | 330.00 ± 487.00 | 335.00 ± 472.00 | 0.95 |
| **CK** (**U/L**) | | 80.50 ± 74.70 | 78.30 ± 159.00 | 0.91 |
| **LDH** (**U/L**) | | 220.00 ± 91.30 | 220.00 ± 97.50 | 0.99 |
| **Echocardiogram results** | |  |  |  |
| -Aortaascendens (mm) | | 2.77 ± 0.41 | 2.70 ± 0.35 | 0.26 |
| -Left atrium (mm) | | 3.85 ± 0.83 | 3.78 ± 0.91 | 0.64 |
| -Left ventricle (mm) | | 4.68 ± 0.94 | 4.41 ± 0.81 | 0.07 |
| -Interventricular septum thickness (mm) | | 0.92 ± 0.51 | 0.86 ± 0.15 | 0.40 |
| -Right atrium (mm) | | 4.37 ± 0.89 | 4.52 ± 0.91 | 0.32 |
| -Reft ventricle (mm) | | 4.25 ± 0.84 | 4.11 ± 1.05 | 0.35 |
| -Pulmonary artery (mm) | | 3.80 ± 3.65 | 3.45 ± 2.57 | 0.53 |
| -Left ventricle fraction shortening (%) | | 33.00 ± 7.53 | 35.3 ± 5.96 | 0.05 |
| -Left ventricular ejection fraction (%) | | 62.40 ± 8.45 | 63.8 ± 7.95 | 0.30 |
| -A-wave of the mitral valve spectrum (m/s) | | 0.94± 0.60 | 1.16 ± 0.97 | 0.07 |
| -Left ventricular outflow tract (m/s) | | 0.92 ± 0.27 | 1.03 ± 0.88 | 0.21 |
| -Aortic valve (m/s) | | 1.39 ± 0.50 | 1.36 ± 0.47 | 0.68 |
| -Pulmonary valve (m/s) | | 1.33 ± 0.62 | 1.18 ± 0.44 | 0.11 |
| Degree of mitral valve regurgitation **( n, %)** | |  |  | 0.57 |
| -None | | 45 (86.54%) | 97 (82.20%) |  |
| -A small amount | | 3 (5.77%) | 13 (11.02%) |  |
| -Medium | | 2 (3.85%) | 6 (5.08%) |  |
| -A large amount | | 2 (3.85%) | 2 (1.69%) |  |
| **Degree of tricuspid regurgitation** **( n, %)** | |  |  | 0.43 |
| -None | | 6 (11.54%) | 14 (11.86%) |  |
| -A small amount | | 15 (28.85%) | 45 (38.14%) |  |
| -Medium | | 18 (34.62%) | 41 (34.75%) |  |
| -A large amount | | 13 (25.00%) | 18 (15.25%) |  |
| **Degree of aortic valve regurgitation ( n, %)** | |  |  | 0.34 |
| -None | | 50 (96.15%) | 105 (88.98%) |  |
| -A small amount | | 2 (3.85%) | 4 (3.39%) |  |
| -Medium | | 0 (0.00%) | 4 (3.39%) |  |
| -A large amount | | 0 (0.00%) | 5 (4.24%) |  |
| **Degree of pulmonary valve regurgitation ( n, %)** | |  |  | 0.83 |
| -None | | 37 (71.15%) | 83 (70.34%) |  |
| -A small amount | | 9 (17.31%) | 25 (21.19%) |  |
| -Medium | | 5 (9.62%) | 7 (5.93%) |  |
| -A large amount | | 1 (1.92%) | 3 (2.54%) |  |
| **ICU admission** **( n, %)** | |  |  | 0.26 |
| -No | | 40 (76.92%) | 79 (66.95%) |  |
| -Yes | | 12 (23.08%) | 39 (33.05%) |  |
| **Blood loss (ml)** | | 348.00 ±690.00 | 251.00 ±164.00 | 0.32 |
| **Anesthesia methods ( n, %)** | |  |  | 0.88 |
| -No anesthesia | | 13 (25.00%) | 27 (22.88%) |  |
| -Intraspinal anesthesia | | 26 (50.00%) | 64 (54.24%) |  |
| -General anesthesia | | 13 (25.00%) | 27 (22.88%) |  |

NYHA, New York Heart Association. PAH, pulmonary arterial hypertension. ECG, Electrocardiogram. SpO2, oxyhemoglobin saturation. TBIL, total bilirubin. ALB, albumin. WBC, white blood cell. Hb, hemoglobin. Hct， hematocrit. RBC, red blood cell. PLT, platelet count. TT, thrombin time. FIB, fibrinogen. APTT, activated partial thromboplastin time. INR, activated partial thromboplastin time. PT, prothrombin time. D-D, D-dimer. BNP, B-type natriuretic peptide. CK, Creatine Kinase. LDH, lactate dehydrogenase.

**Table S2 Comparison of the general situation of maternal cardiovascular complications in pregnant patients with PAH and those without maternal cardiovascular complications**

|  | Control group (n=145) | Complication group (n=25) | t/ U/X^2^ | P-value |
| --- | --- | --- | --- | --- |
| **Age（years）** | 28.37 ± 4.55 | 29.4 4± 5.31 | -1.06 | 0.29 |
| **Gravidity (number of pregnancies)** | 2.00 (1.00, 3.00) | 1.00 (1.00, 4.00) | 1746.50 | 0.76 |
| **Parity (number of deliveries)** | 0.00 (0.00, 1.00) | 0.00 (0.00, 1.00) | 1633.00 | 0.36 |
| **Nulliparity ( n, %)** | 90 (62.10%) | 14 (56.00%) | 0.33 | 0.57 |
| **Height (cm)** | 160.38 ± 4.40 | 158.88 ± 4.44 | 1.57 | 0.12 |
| **Weight (Kg)** | 61.54 ± 11.20 | 59.40 ± 11.26 | 0.88 | 0.379 |
| **Gestational age at termination (days)** | 256.00 (180.55，265.00) | 238.00 (198.50，258.50) | 1551.50 | 0.25 |
| **PAH classification ( n, %)** |  |  | 7.72 | 0.01^*^ |
| -Mild | 63 (43.45%) | 6 (24.00%) |  |  |
| -Moderate | 33 (22.76%) | 3 (12.00%) |  |  |
| -Severe | 49 (33.79%) | 16 (64.00%) |  |  |

^*^Pearson correlation coefficient, the results showed that R=0.2, P (Approx.Sig.) <0.05, indicating that there is a low positive correlation between the occurrence of cardiovascular complications and PAH classification.

**Table S3 Comparison of the past medical history of maternal cardiovascular complications in pregnant patients with PAH and those without maternal cardiovascular complications**

|  | Control group (n=145) | Complication group (n=25) | X^2^ | P-value |
| --- | --- | --- | --- | --- |
| **Unexplained or idiopathic PAH ( n, %)** | 6 (4.13%) | 1 (4.00%) | 0.70 | 0.68^*^ |
| -Unexplained PAH | 4 | 1 |  |  |
| -Idiopathic PAH | 2 | 0 |  |  |
| **Connective tissue disease ( n, %)** | 20 (13.79%) | 8 (32.00%) | 6.85 | 0.77 |
| -Rheumatic heart disease | 14 | 7 |  |  |
| -Systemic lupus erythematosus | 5 | 1 |  |  |
| -Other types of connective tissue diseases | 1 | 0 |  |  |
| **Congenital heart disease ( n, %)** | 107 (73.79%) | 12 (48.00%) | 10.24 | 0.33 |
| -Coronary sinus defect | 1 | 0 |  |  |
| -Atrial septal defect | 58 | 5 |  |  |
| -Ventricular septal defect | 28 | 4 |  |  |
| -Complex congenital heart disease | 12 | 2 |  |  |
| -Patent ductus arteriosus | 3 | 1 |  |  |
| -Patent foramen ovale | 2 | 0 |  |  |
| -Papillary muscle dysplasia | 1 | 0 |  |  |
| -Noncompaction of the ventricular myocardium | 2 | 0 |  |  |
| **Valvular heart disease ( n, %)** | 9 (6.21%) | 1 (4.00%) | 0.19 | 1.00^*^ |
| **Complications during pregnancy ( n, %)** | 3 (2.07%) | 3 (12.00%） | 6.49 | 0.04^*^ |
| -Severe preeclampsia | 1 | 2 |  |  |
| -Peripartum cardiomyopathy | 2 | 1 |  |  |
| **Detection of PAH( n, %)** |  |  | 3.31 | 0.13 |
| -Pre-pregnancy | 69 (47.59%) | 7 (28.00%) |  |  |
| -After pregnancy | 76 (52.41%) | 18 (72.00%) |  |  |
| **Presence of Cardiac Structural Changes** **( n, %)** |  |  | 2.25 | 0.13 |
| -None | 42 (28.97%) | 11 (44.00%) |  |  |
| -Present | 103 (71.03%) | 14 (56.00%) |  |  |
| **History of treatment for heart disease** **( n, %)** |  |  | 3.70 | 0.07^*^ |
| -None | 108 (74.48%) | 23 (92.00%) |  |  |
| -Present | 37 (25.52%) | 2 (8.00%) |  |  |
| **History of cardiac-related surgery before pregnancy** |  |  | 8.44 | 0.001^*^ |
| -None | 107 (73.79%) | 25 (100.00%) |  |  |
| -Present | 38 (26.21%) | 0 (0.00%) |  |  |

^*^ Fisher test

**Table S4 Comparison of pregnancy complications and comorbidities in pregnant women with PAH with and without maternal cardiovascular complications**

|  | Control group (n=145) | Complication group (n=25) | X^2^ | P-value |
| --- | --- | --- | --- | --- |
| **Upper respiratory tract infection (cases, %)** | 3 (2.07%) | 1 (4.00%) | 0.35 | 0.47^*^ |
| **Hypertensive disorders during pregnancy (cases, %)** | 11 (7.59%) | 3 (12.00%) | 2.54 | 0.64 |
| -severe preeclampsia | 3 | 1 |  |  |
| -Preeclampsia | 1 | 1 |  |  |
| -Hypertension during pregnancy | 6 | 1 |  |  |
| -HELLP syndrome | 1 | 0 |  |  |
| **Intrahepatic cholestasis of pregnancy (cases, %)** | 2 (1.38%) | 1 (4.00%) | 0.85 | 0.38^*^ |
| **Gestational diabetes (cases, %)** | 8 (5.52%) | 0 (0.00%) | 1.45 | 0.61^*^ |
| **Thrombocytopenia (cases, %)** | 1 (0.69%) | 1 (4.00%) | 2.01 | 0.27^*^ |
| **Hypothyroidism (cases, %)** | 3 (2.07%) | 0 (0.00%) | 0.53 | 1.00^*^ |
| **Fetal appendage abnormalities (cases, %)** | 9 (6.21%) | 3 (12.00%) | 9.06 | 0.17 |
| -Oligohydramnios | 1 | 1 |  |  |
| -Placental abruption | 2 | 0 |  |  |
| -Placenta previa | 2 | 0 |  |  |
| -Placental adhesion | 1 | 0 |  |  |
| -Premature rupture of membranes | 3 | 1 |  |  |
| -Placenta accreta | 0 | 1 |  |  |

^*^ Fisher test

**Table S5** **Comparison of NYHA classification in pregnant women with PAH with and without maternal cardiovascular complications**

|  | Control group (n=145) | Complication group (n=25) | X^2^ | P-value |
| --- | --- | --- | --- | --- |
| **NYHA classification** |  |  | 24.09 | 0.00^*^ |
| -Class I | 7 (4.83%) | 1 (4.00%) |  |  |
| -Class II | 98 (67.59%) | 5 (20.00%) |  |  |
| -Class III | 37 (25.52%) | 16 (64.00%) |  |  |
| -Class IV | 3 (2.07%) | 3 (12.00%) |  |  |

^*^ The Pearson correlation coefficient indicates a moderate positive correlation between the incidence of cardiovascular complications and NYHA heart function classification, with R = 0.337 and a p-value (Approx. Sig.) of less than 0.001. NYHA, New York Heart Association.

**Table S6 Comparison of echocardiographic outcomes of pregnant women with PAH with and without maternal cardiovascular complications**

|  | Control group b (n=145) | Complication group (n=25) | t/ U/X^2^ | P-value |
| --- | --- | --- | --- | --- |
| **Aortaascendens (mm)** | 2.72 ± 0.36 | 2.76 ± 0.44 | -0.51 | 0.61 |
| **Left atrium (mm)** | 3.74 ± 0.84 | 4.15 ± 1.09 | -2.14 | 0.03 |
| **Left ventricle (mm)** | 4.30 (4.00, 4.85) | 4.30 (3.55, 5.40) | 1735.0- | 0.73 |
| **Interventricular septum thickness (mm)** | 0.90 (0.80, 1.00) | 0.80 (0.75, 0.90) | 1380.0- | 0.05 |
| **Right atrium (mm)** | 4.40 (3.90, 5.00) | 4.50 (4.10, 5.25) | 1675.50 | 0.55 |
| **Reft ventricle (mm)** | 4.20 (3.60, 4.75) | 4.20 (3.80, 5.20) | 1585.0 | 0.32 |
| **Pulmonary artery (mm)** | 3.57 ± 3.16 | 3.48 ± 0.77 | 0.13 | 0.90 |
| **Left ventricle fraction shortening (%)** | 34.74 ± 6.35 | 33.88 ± 7.75 | 0.61 | 0.55 |
| **Left ventricular ejection fraction (%)** | 63.50 ± 6.35 | 62.64 ± 9.82 | 0.49 | 0.68 |
| **A-wave of the mitral valve spectrum (m/s)** | 0.80 (0.60, 1.00) | 0.90 (0.60, 2.90) | 1451.50 | 0.11 |
| **Left ventricular outflow tract (m/s)** | 0.80 (0.70, 1.00) | 0.90 (0.70, 1.00) | 1677.50 | 0.55 |
| **Aortic valve (m/s)** | 1.30 (1.10, 1.50) | 1.20 (1.00, 1.75) | 1709.00 | 0.65 |
| **Pulmonary valve (m/s)** | 1.20 (0.90, 1.40) | 1.00 (0.70, 1.20) | 1156.50 | 0.004 |
| **Degree of mitral valve regurgitation ( n, %)** |  |  | 6.40 | 0.06 |
| -None | 125 (86.21%) | 17 (68.00%) |  |  |
| -A small amount | 12 (8.28%) | 4 (16.00%) |  |  |
| -Medium | 5 (3.45%) | 3 (12.00%) |  |  |
| -A large amount | 3 (2.07%) | 1 (4.00%) |  |  |
| **Degree of tricuspid regurgitation** **( n, %)** |  |  | 4.47 | 0.22 |
| -None | 18 (12.42%) | 2 (8.00%) |  |  |
| -A small amount | 54 (37.24%) | 6 (24.00%) |  |  |
| -Medium | 50 (34.49%) | 9 (36.00%) |  |  |
| -A large amount | 23 (15.86%) | 8 (32.00%) |  |  |
| **Degree of aortic valve regurgitation ( n, %)** |  |  | 9.75 | 0.002^*^ |
| -None | 136 (93.79%) | 19 (76.00%) |  |  |
| -A small amount | 4 (2.76%) | 2 (8.00%) |  |  |
| -Medium | 3 (2.07%) | 1 (4.00%) |  |  |
| -A large amount | 2 (1.38%) | 4 (16.00%) |  |  |
| **Degree of pulmonary valve regurgitation ( n, %)** |  |  | 1.85 | 0.61 |
| -None | 104 (71.72%) | 16 (64.00%) |  |  |
| -A small amount | 27 (18.62%) | 7 (28.00%) |  |  |
| -Medium | 10 (6.90%) | 2 (8.00%) |  |  |
| -A large amount | 4 (2.76%) | 0 (0.00%) |  |  |

*The Pearson correlation coefficient indicates a slight positive correlation between the occurrence of cardiovascular complications and the severity of aortic valve regurgitation, with R = 0.240 and a p-value (Approx. Sig.) of 0.003, which is less than 0.05.

**Table S7 Comparison of ECG results in pregnant women with PAH with and without maternal cardiovascular complications**

|  | Control group (n=145) | Complication group (n=25) | X^2^ | P-value |
| --- | --- | --- | --- | --- |
| **ECG results** **( n, %)** |  |  |  |  |
| -Sinus tachycardia | 22 (15.17%) | 6 (24.00%) | 1.21 | 0.27 |
| -ST-T changes | 18 (12.42%) | 7 (28.00%) | 4.13 | 0.04 |
| -T wave changes | 14 (9.66%) | 4 (16.00%) | 0.91 | 0.31* |
| -Left ventricular high voltage | 4 (2.76%) | 3 (12.00%) | 4.61 | 0.07* |
| -Right ventricular high voltage | 4 (2.76%) | 3 (12.00%) | 4.61 | 0.07* |
| -Right axis deviation | 20 (13.79%) | 6 (24.00%) | 1.72 | 0.19 |
| -Left atrial hypertrophy | 14 (9.66%) | 7 (28.00%) | 6.63 | 0.01 |
| -Right atrial hypertrophy | 2 (1.38%) | 0 (0.00%) | 0.35 | 1.00* |
| -Right ventricular hypertrophy | 22 (15.17%) | 6 (24.00%) | 1.21 | 0.27 |
| -Clockwise rotation | 10 (6.90%) | 4 (16.00%) | 2.34 | 0.13* |
| -Atrial fibrillation | 0 (0.00%) | 1 (4.00%) | 5.83 | 0.15* |
| -Abnormal Q wave | 2 (1.38%) | 5 (20.00%) | 18.73 | 0.001* |
| -Ventricular premature beats | 7 (4.83%) | 2 (8.00%) | 0.43 | 0.62* |
| -Incomplete right bundle branch block | 35 (24.14%) | 8 (32.00%) | 0.70 | 0.40 |
| -Complete right bundle branch block | 10 (6.90%) | 1 (4.00%) | 0.30 | 1.00* |
| -Paroxysmal supraventricular tachycardia | 1 (0.69%) | 0 (0.00%) | 0.17 | 1.00* |

*Fisher test

**Table S8 Comparison of blood biochemical examination in pregnant women with PAH with and without maternal cardiovascular complications**

|  | Control group (n=145) | Complication group (n=25) | t/U | P-value |
| --- | --- | --- | --- | --- |
| **SpO_2_（%）** | 98.00 (97.00, 99.00) | 97.00 (95.00, 98.00) | 1111.00 | 0.001 |
| **TBIL（umol/L）** | 8.50 (6.60, 11.25) | 12.80 (9.60, 18.40) | 945.00 | 0.00 |
| **ALB（g/L）** | 32.65 ± 4.07 | 28.33 ± 5.45 | 3.79 | 0.001 |
| **WBC（G/L）** | 8.51 ±2.55 | 8.17 ± 2.28 | 0.58 | 0.56 |
| **Hb（g/L）** | 113.00 (99.50, 122.60) | 106.00 (98.00, 122.50) | 1669.00 | 0.53 |
| **Hct（%）** | 34.20 (31.55, 37.20) | 33.40 (30.05, 38.50) | 1760.00 | 0.82 |
| **RBC（T/L）** | 3.81 (3.52, 4.11) | 3.67 (3.27, 4.17) | 1553.00 | 0.25 |
| **Plt（G/L）** | 194.96 ± 58.69 | 190.00 ± 62.47 | 0.39 | 0.70 |
| **TT（s）** | 16.30 (15.50, 17.10) | 16.80 (15.95, 17.35) | 1520.50 | 0.20 |
| **FIB（g/L）** | 4.15 (3.37, 4.80) | 4.03 (3.44, 5.55) | 1777.00 | 0.88 |
| **APTT（s）** | 33.64 ± 6.72 | 34.31 ± 3.56 | -0.49 | 0.63 |
| **INR** | 0.97 (0.92, 1.07) | 0.96 (0.91, 1.10) | 1744.50 | 0.77 |
| **PT（s）** | 13.06 ± 2.64 | 12.86 ± 1.41 | 0.38 | 0.71 |
| **D-D（mg/L）** | 0.89 (0.58, 1.68) | 1.12 (0.78, 1.88) | 1468.00 | 0.13 |
| **BNP（pg/ml）** | 100.80 (30.50, 344.00) | 512.30 (164.10, 917.50) | 928.50 | 0.00 |
| **CK（U/L）** | 48.00 (27.00, 75.00) | 42.00 (26.00, 88.00) | 1808.00 | 0.98 |
| **LDH（U/L）** | 213.90 ± 90.18 | 257.09 ± 116.51 | -2.11 | 0.04 |

SpO_2_, oxyhemoglobin saturation. TBIL, total bilirubin. ALB, albumin. WBC, white blood cell. Hb, hemoglobin. Hct, hematocrit. RBC, red blood cell. PLT, platelet count. TT, thrombin time. FIB, fibrinogen. APTT, activated partial thromboplastin time. INR, activated partial thromboplastin time. PT, prothrombin time. D-D, D-dimer. BNP, B-type natriuretic peptide. CK, Creatine Kinase. LDH, lactate dehydrogenase.

**Table S9** **Comparison of pregnancy outcomes in patients with pregnancy complicated by PAH with and without maternal cardiovascular complications**

|  | Control group (n=145) | Complication group (n=25) | X^2^ | P-value |
| --- | --- | --- | --- | --- |
| **Methods of terminating pregnancy ( n, %)** |  |  | 7.32 | 0.12 |
| -Iatrogenic abortion in the first trimester | 28 (19.31%) | 1 (4.00%) |  |  |
| -Induced labor in the second trimester | 5 (3.45%) | 2 (8.00%) |  |  |
| -Vaginal delivery | 2 (2.38%) | 1 (4.00%) |  |  |
| -Cesarean section to remove the fetus | 6 (4.14%) | 3 (12.00%) |  |  |
| -Cesarean delivery | 104 (71.72%) | 18 (72.00%) |  |  |
| **ICU admission** **( n, %)** |  |  | 20.15 | 0.00 |
| -No | 111 (76.55%) | 8 (32.00%) |  |  |
| -Yes | 34 (23.45%) | 17 (68.00%) |  |  |
| **Blood loss (ml)** | 200.00 (200.00, 300.00) | 200.00 (200.00, 300.00) | 1608.00 | 0.35 |
| **Anesthesia methods ( n, %)** |  |  | 2.54 | 0.28 |
| -No anesthesia | 35 (24.14%) | 5 (20.00%) |  |  |
| -Intraspinal anesthesia | 79 (54.48%) | 11 (44.00%) |  |  |
| -General anesthesia | 31 (21.38%) | 9 (36.00%) |  |  |

**Table S10 Comparison of neonatal outcomes in pregnancies complicated by PAH with and without maternal cardiovascular complications**

|  | Control group (n=112) | Complication group (n=22) | X^2^/U | P-value |
| --- | --- | --- | --- | --- |
| Apgar Score |  |  |  |  |
| -1 minute | 8 (8, 8) | 8 (6, 8) | 946.00 | 0.06 |
| -5 minute | 9 (9, 9) | 9 (7, 9) | 922.50 | 0.04 |
| Admission to NICU (n, %) | 33 (29.46%) | 12 (54.55%) | 5.19 | 0.02 |
| Birth weight of newborn (Kg) | 2750 (2265, 3050) | 2150 (1600, 2650) | 695.00 | 0.001 |
| Low birth weitht infants (n, %) | 28 (25.00%) | 4 (18.19%) | 0.47 | 0.49 |
| Preterm (n, %) | 19 (16.96%) | 6 (27.27%) | 1.29 | 0.26 |
| Intrauterine Growth Restriction (n, %) | 6 (5.36%) | 0 (0.00%) | 1.23 | 0.27 |
| Fetal Distress (n, %) | 6 (5.36%) | 6 (27.27%) | 10.83 | 0.001 |
| Intrauterine fetal death (n, %) | 6 (5.36%) | 3 (13.64%) | 2.01 | 0.16 |

^*^ After excluding 36 cases of induced abortions, a total of 134 cases were included in the study.

**Table S11** **Comparison of clinical data characteristics between the training set and validation set of the prediction model for adverse neonatal outcomes in pregnant patients with pulmonary hypertension**

|  | Validation set (n=41) | Training Set (n=93) | P-value |
| --- | --- | --- | --- |
| **Age（years）** | 29.00 ± 5.00 | 28.40 ± 4.80 | 0.49 |
| **Gravidity** | 1.93 ± 1.42 | 2.10 ± 1.38 | 0.52 |
| **Parity** | 0.34 ± 0.62 | 0.49 ± 0.65 | 0.20 |
| **Height (cm)** | 160.00 ± 4.13 | 161.00 ± 4.35 | 0.37 |
| **Weight (Kg)** | 63.70 ± 9.27 | 64.40 ± 10.70 | 0.67 |
| **Gestational age at termination (days)** | 253.00 ± 25.40 | 251.00 ± 25.50 | 0.81 |
| **Methods of terminating pregnancy ( n, %)** |  |  | 0.66 |
| -Vaginal delivery | 0 (0.00%) | 3 (3.23%) |  |
| -Cesarean section to remove the fetus | 2 (4.88%) | 7 (7.53%) |  |
| -Cesarean delivery | 39 (95.12%) | 83 (89.24%) |  |
| **PAH classification ( n, %)** |  |  | 0.33 |
| -Mild | 21 (51.22%) | 44 (47.31%) |  |
| -Moderate | 6 (14.63%) | 24 (25.81%) |  |
| -Severe | 14 (34.15%) | 25 (26.88%) |  |
| **Cause of PAH** **( n, %)** |  |  | 0.91 |
| -Unexplained or idiopathic PAH | 2 (4.88%) | 3 (3.23%) |  |
| -Connective tissue disease | 8 (19.51%) | 19 (20.43%) |  |
| -Congenital heart disease | 27 (65.85%) | 61 (65.59%) |  |
| -Valvular heart disease | 3 (7.32%) | 5 (5.38%) |  |
| -Complications during pregnancy | 1 (2.44%) | 5 (5.38%) |  |
| **NYHA classification ( n, %)** |  |  | 0.81 |
| -Class I | 2 (4.88%) | 3 (3.23%) |  |
| -Class II | 23 (56.10%) | 57 (61.29%) |  |
| -Class III | 14 (34.15%) | 30 (32.26%) |  |
| -Class IV | 2 (4.88%) | 3 (3.23%) |  |
| **Detection of PAH( n, %)** |  |  | 0.08 |
| -Pre-pregnancy | 22 (53.66%) | 33 (35.48%) |  |
| -After pregnancy | 19 (46.34%) | 60 (64.52%) |  |
| **Presence of Cardiac Structural Changes** **( n, %)** |  |  | 0.82 |
| -None | 13 (31.71%) | 33 (35.48%) |  |
| -Present | 28 (68.29%) | 60 (64.52%) |  |
| **History of treatment for heart disease** **( n, %)** |  |  | 0.45 |
| -None | 28 (68.29%) | 71 (76.34%) |  |
| -Present | 13 (31.71%) | 22 (23.66%) |  |
| **History of cardiac-related surgery before pregnancy** |  |  | 0.64 |
| -None | 29 (70.73%) | 71 (76.34%) |  |
| -Present | 12 (29.27%) | 22 (23.66%) |  |
| **ECG results** **( n, %)** |  |  |  |
| -Sinus tachycardia | 7 (17.07%) | 19 (20.43%) | 0.83 |
| -ST-T changes | 7 (17.07%) | 10 (10.75%) | 0.47 |
| -T wave changes | 6 (14.63%) | 9 (9.68%) | 0.39 |
| -Left ventricular high voltage | 2 (4.88%) | 3 (3.23%) | 0.64 |
| -Right ventricular high voltage | 2 (4.88%) | 3 (3.23%) | 0.64 |
| -Right axis deviation | 8 (19.51%) | 13 (14.00%) | 0.58 |
| -Left atrial hypertrophy | 8 (19.51%) | 13 (14.00%) | 0.58 |
| -Right atrial hypertrophy | 1 (2.44%) | 1 (1.08%) | 0.52 |
| -Right ventricular hypertrophy | 4 (9.76%) | 11 (11.83%) | 1.00 |
| -Clockwise rotation | 5 (12.20%) | 6 (6.45%) | 0.31 |
| -Atrial fibrillation | 0 (0.00%) | 1 (1.08%) | 1.00 |
| -Abnormal Q wave | 2 (4.88%) | 4 (4.30%) | 1.00 |
| -Ventricular premature beats | 2 (4.88%) | 4 (4.30%) | 1.00 |
| -Incomplete right bundle branch block | 12 (29.27%) | 24 (25.81%) | 0.84 |
| -Complete right bundle branch block | 3 (7.32%) | 6 (6.45%) | 1.00 |
| **SpO_2_（%）** | 97.00 ± 3.26 | 96.10 ± 10.30 | 0.41 |
| **TBIL（umol/L）** | 9.51 ± 4.06 | 10.90 ± 6.93 | 0.15 |
| **ALB（g/L）** | 32.60 ± 4.06 | 31.70 ± 4.41 | 0.21 |
| **WBC（G/L）** | 8.95 ± 2.28 | 8.72 ± 2.60 | 0.62 |
| **Hb（g/L）** | 107.00 ± 22.6 | 109.00 ± 16.5 | 0.63 |
| **Hct（%）** | 33.40 ± 4.17 | 32.90 ± 5.63 | 0.57 |
| **RBC（T/L）** | 3.79 ± 0.50 | 3.71 ± 0.58 | 0.42 |
| **Plt（G/L）** | 208.00 ± 59.20 | 195.00 ± 59.5 | 0.24 |
| **TT（s）** | 19.90 ± 23.90 | 16.10 ± 1.85 | 0.32 |
| **FIB（g/L）** | 5.44 ± 5.12 | 4.29 ± 0.97 | 0.16 |
| **APTT（s）** | 31.90 ± 5.81 | 32.60 ± 5.31 | 0.50 |
| **INR** | 1.55 ± 2.52 | 1.01 ± 0.26 | 0.19 |
| **PT（s）** | 12.70 ± 3.01 | 12.80 ± 2.56 | 0.88 |
| **D-D（mg/L）** | 1.67 ± 2.39 | 2.11 ± 7.50 | 0.61 |
| **BNP（pg/ml）** | 346.00 ± 485.00 | 366.00 ± 526.00 | 0.83 |
| **CK（U/L）** | 109.00 ± 249.00 | 67.70 ± 65.30 | 0.30 |
| **LDH（U/L）** | 230.00 ± 97.30 | 220.00 ± 96.00 | 0.58 |
| **Echocardiogram results** |  |  |  |
| -Aortaascendens (mm) | 2.74 ± 0.37 | 2.74 ± 0.37 | 0.98 |
| -Left atrium (mm) | 3.98 ± 0.97 | 3.95 ± 0.85 | 0.86 |
| -Left ventricle (mm) | 4.67 ± 0.95 | 4.58 ± 0.82 | 0.59 |
| -Interventricularseptum thickness (mm) | 0.89 ± 0.15 | 0.90 ± 0.39 | 0.87 |
| -Right atrium (mm) | 4.63 ± 0.92 | 4.41 ± 0.92 | 0.21 |
| -Reft ventricle (mm) | 4.19 ± 1.07 | 4.05 ± 1.05 | 0.49 |
| -Pulmonary artery (mm) | 3.17 ± 0.73 | 3.42 ± 2.76 | 0.42 |
| -Left ventricle ftactionshortening (%) | 34.30 ± 7.07 | 34.90 ± 6.89 | 0.66 |
| -Left ventricular ejection fiaction (%) | 62.50 ± 5.82 | 63.40 ± 9.65 | 0.52 |
| -A-wave of the mitral valve spectrum (m/s) | 1.15 ± 0.90 | 1.20 ± 0.93 | 0.76 |
| -Left ventricular outflow tract (m/s) | 1.04 ± 0.77 | 0.98 ± 0.72 | 0.65 |
| -Aorticvalve (m/s) | 1.38 ± 0.49 | 1.38 ± 0.49 | 1.00 |
| -Pulmonary valve (m/s) | 1.27 ± 0.51 | 1.22 ± 0.54 | 0.56 |
| Degree of mitral valve regurgitation **( n, %)** |  |  | 0.40 |
| -None | 31 (75.6%) | 79 (84.9%) |  |
| -A small amount | 6 (14.6%) | 7 (7.53%) |  |
| -Medium | 2 (4.88%) | 5 (5.38%) |  |
| -A large amount | 2 (4.88%) | 2 (2.15%) |  |
| **Degree of tricuspid regurgitation** **( n, %)** |  |  | 0.10 |
| -None | 1 (2.44%) | 13 (14.0%) |  |
| -A small amount | 13 (31.7%) | 36 (38.7%) |  |
| -Medium | 17 (41.5%) | 30 (32.3%) |  |
| -A large amount | 10 (24.4%) | 14 (15.1%) |  |
| **Degree of aortic valve regurgitation ( n, %)** |  |  | 0.35 |
| -None | 38 (92.7%) | 82 (88.2%) |  |
| -A small amount | 0 (0.00%) | 6 (6.45%) |  |
| -Medium | 1 (2.44%) | 2 (2.15%) |  |
| -A large amount | 2 (4.88%) | 3 (3.23%) |  |
| **Degree of pulmonary valve regurgitation ( n, %)** |  |  | 0.5 |
| -None | 29 (70.7%) | 72 (77.4%) |  |
| -A small amount | 9 (22.0%) | 16 (17.2%) |  |
| -Medium | 3 (7.32%) | 3 (3.23%) |  |
| -A large amount | 0 (0.00%) | 2 (2.15%) |  |
| **ICU admission** **( n, %)** |  |  | 1.000 |
| -No | 27 (65.9%) | 62 (66.7%) |  |
| -Yes | 14 (34.1%) | 31 (33.3%) |  |
| **Blood loss (ml)** | 262.00 ± 171.00 | 343.00 ± 515.00 | 0.18 |
| **Anesthesia methods ( n, %)** |  |  | 0.19 |
| -No anesthesia | 0 (0.00%) | 5 (5.38%) |  |
| -Intraspinal anesthesia | 31 (75.6%) | 58 (62.4%) |  |
| -General anesthesia | 10 (24.4%) | 30 (32.3%) |  |

NYHA, New York Heart Association. PAH, pulmonary arterial hypertension. ECG, Electrocardiogram. SpO_2_, oxyhemoglobin saturation, TBIL, total bilirubin. ALB, albumin. WBC, white blood cell. Hb, hemoglobin. Hct, hematocrit. RBC, red blood cell. PLT, platelet count. TT, thrombin time. FIB, fibrinogen. APTT, activated partial thromboplastin time. INR, activated partial thromboplastin time. PT, prothrombin time. D-D, D-dimer. BNP, B-type natriuretic peptide. CK, Creatine Kinase. LDH, lactate dehydrogenase.

**Table S12 Comparison of general conditions between pregnant patients complicated by PAH with and without adverse neonatal outcomes**

|  | Control group (n=83) | Adverse outcome group (n=51) | t/ U/X^2^ | P-value |
| --- | --- | --- | --- | --- |
| **Age（years）** | 24.18 ± 4.92 | 28.00 ± 22.54 | -0.97 | 0.37 |
| **Gravidity** | 2.00 (1.00, 3.00) | 2.00 (1.00, 3.00) | 2035.50 | 0.69 |
| **Parity** | 0.00 (0.00, 1.00) | 0.00 (1.00, 3.00) | 2115.00 | 0.99 |
| **Height (cm)** | 18.00 ± 4.24 | 19.03 ± 4.36 | -0.74 | 0.46 |
| **Weight (Kg)** | 100.05 ± 10.00 | 106.10 ± 30 | 2.15 | 0.03 |
| **Gestational age at termination (days)** | 263.00 (248.00, 270.00) | 249.00 (218.00, 256.00) | 807.50 | 0.00 |
| **PAH classification ( n, %)** |  |  | 5.86 | 0.05 |
| -Mild | 44 (53.0%) | 21 (32.3%) |  |  |
| -Moderate | 21 (25.3%) | 9 (17.6%) |  |  |
| -Severe | 18 (21.7%) | 21 (41.2%) |  |  |

PAH, pulmonary arterial hypertension.

**Table S13 Comparison of the cause of PAH between pregnant patients complicated by PAH with and without adverse neonatal outcomes**

|  | Control group (n=83) | Adverse outcome group (n=51) | X^2^ | P-value |
| --- | --- | --- | --- | --- |
| **Unexplained or idiopathic PAH ( n, %)** | 3 (3.61%) | 2 (3.92%) | 0.008 | 1.00^*^ |
| -Unexplained PAH | 3 | 2 |  |  |
| -Idiopathic PAH | 0 | 0 |  |  |
| **Connective tissue disease ( n, %)** | 15 (18.07%) | 12 (23.53%) | 2.22 | 0.53 |
| -Rheumatic heart disease | 11 | 9 |  |  |
| -Systemic lupus erythematosus | 3 | 3 |  |  |
| -Other types of connective tissue diseases | 1 | 0 |  |  |
| **Congenital heart disease ( n, %)** | 57 (68.67%) | 31 (60.78%) | 10.28 | 0.32 |
| -Coronary sinus defect | 0 | 1 |  |  |
| -Atrial septal defect | 35 | 14 |  |  |
| -Ventricular septal defect | 12 | 9 |  |  |
| -Complex congenital heart disease | 6 | 4 |  |  |
| -Patent ductus arteriosus | 1 | 1 |  |  |
| -Patent foramen ovale | 1 | 1 |  |  |
| -Papillary muscle dysplasia | 1 | 0 |  |  |
| -Noncompaction of the ventricular myocardium | 1 | 1 |  |  |
| **Valvular heart disease ( n, %)** | 5 (6.02%) | 3 (5.88%) | 0.001 | 0.97^*^ |
| **Complications during pregnancy ( n, %)** | 3 (3.61%) | 3 (5.88%) | 1.09 | 0.80^*^ |
| -Severe preeclampsia | 1 | 2 |  |  |
| -Peripartum cardiomyopathy | 2 | 1 |  |  |

*Fisher test

**Table S14 Comparison of the past medical history of maternal cardiovascular complications in pregnant patients with PAH and those without adverse neonatal outcomes**

|  | Control group (n=83) | Adverse outcome group (n=51) | X^2^ | P-value |
| --- | --- | --- | --- | --- |
| **Detection of PAH( n, %)** |  |  | 1.13 | 0.37 |
| -Pre-pregnancy | 37 (44.58%) | 18 (35.29%) |  |  |
| -After pregnancy | 46 (55.42%) | 33 (64.71%) |  |  |
| **Presence of Cardiac Structural Changes** **( n, %)** |  |  | 0.32 | 0.58 |
| -None | 30 (36.14%) | 16 (31.37%) |  |  |
| -Present | 53 (63.86%) | 35 (68.63%) |  |  |
| **History of treatment for heart disease** **( n, %)** |  |  | 0.08 | 0.84 |
| -None | 62 (74.70%) | 37 (72.55%) |  |  |
| -Present | 21 (25.30%) | 14 (27.45%) |  |  |
| **History of cardiac-related surgery before pregnancy** |  |  | 0.15 | 0.84 |
| -None | 61 (73.49%) | 39 (76.47%) |  |  |
| -Present | 22 (26.51%) | 12 (23.53%) |  |  |

**Table S15 Comparison of pregnancy complications and comorbidities in pregnant women with PAH with and without adverse neonatal outcomes**

|  | Control group (n=83) | Adverse outcome group (n=51) | X^2^ | P-value |
| --- | --- | --- | --- | --- |
| **Upper respiratory tract infection (cases, %)** | 2 (2.41%) | 2 (3.92%) | 2.49 | 0.64^*^ |
| **Hypertensive disorders during pregnancy (cases, %)** | 5 (6.02%) | 6 (11.76%) | 4.38 | 0.39 |
| -severe preeclampsia | 1 | 3 |  |  |
| -Preeclampsia | 1 | 1 |  |  |
| -Hypertension during pregnancy | 3 | 4 |  |  |
| -HELLP syndrome | 1 | 0 |  |  |
| **Intrahepatic cholestasis of pregnancy (cases, %)** | 0 (0.00%) | 3 (5.88%) | 4.99 | 0.05^*^ |
| **Gestational diabetes (cases, %)** | 5 (6.02%) | 2 (3.92%) | 0.001 | 1.00^*^ |
| **Thrombocytopenia (cases, %)** | 1 (1.20%) | 1 (1.96%) | 0.123 | 1.00^*^ |
| **Hypothyroidism (cases, %)** | 3 (3.61%) | 0 (0.00%) | 1.89 | 0.29^*^ |
| **Fetal appendage abnormalities (cases, %)** | 5 (6.02%) | 7 (13.73%) | 5.13 | 0.59 |
| -Oligohydramnios | 1 | 1 |  |  |
| -Placental abruption | 1 | 1 |  |  |
| -Placenta previa | 1 | 1 |  |  |
| -Placental adhesion | 1 | 0 |  |  |
| -Premature rupture of membranes | 1 | 3 |  |  |
| -Placenta accreta | 0 | 1 |  |  |

^*^Fisher test

**Table S16** **Comparison of NYHA classification in pregnant women with PAH with and without adverse neonatal outcomes**

|  | Control group (n=83) | Adverse outcome group (n=51) | X^2^ | P-value |
| --- | --- | --- | --- | --- |
| **NYHA classification** |  |  | 4.41 | 0.24 |
| -Class I | 4 (4.82%) | 1 (1.96%) |  |  |
| -Class II | 54 (65.06%) | 26 (51.10%) |  |  |
| -Class III | 22 (26.51%) | 22 (43.14%) |  |  |
| -Class IV | 3 (3.61%) | 2 (3.92%) |  |  |

NYHA, New York Heart Association

**Table S17 Comparison of echocardiographic outcomes of pregnant women with PAH with and without adverse neonatal outcomes**

|  | Control group (n=83) | Adverse outcome group (n=51) | t/ U/X^2^ | P-value |
| --- | --- | --- | --- | --- |
| **Aortaascendens (mm)** | 0.14 ± 0.38 | 0.12 ± 0.34 | 1.53 | 0.13 |
| **Left atrium (mm)** | 3.90 (3.40, 4.60) | 3.80 (3.30, 4.40) | 2081.50 | 0.87 |
| **Left ventricle (mm)** | 4.40 (4.10, 5.20) | 4.30(3.90, 5.00) | 1763.50 | 0.11 |
| **Interventricular septum thickness (mm)** | 0.90 (0.80, 1.00) | 0.90 (0.80, 0.90) | 1890.00 | 0.29 |
| **Right atrium (mm)** | 0.93 ± 0.97 | 0.71 ± 0.84 | -1.40 | 0.16 |
| **Reft ventricle (mm)** | 4.10 (3.60, 4.50) | 4.40 (3.70, 4.90) | 1764.50 | 0.11 |
| **Pulmonary artery (mm)** | 3.00 (2.70, 3.50) | 3.20 (2.60, 3.50) | 1930.00 | 0.39 |
| **Left ventricle fraction shortening (%)** | 35.00 (31.00, 39.00) | 35.00 (31.00, 37.00) | 2050.50 | 0.76 |
| **Left ventricular ejection fraction (%)** | 64.00 (60.00, 69.00) | 64.00 (60.00, 67.00) | 2105.00 | 0.96 |
| **A-wave of the mitral valve spectrum (m/s)** | 0.80 (0.60, 1.20) | 0.80 (0.60, 1.40) | 2009.00 | 0.62 |
| **Left ventricular outflow tract (m/s)** | 0.90 (0.70, 1.10) | 0.80 (0.70, 1.10) | 1759.50 | 0.10 |
| **Aortic valve (m/s)** | 1.30 (1.10, 1.50) | 1.20 (1.00, 1.60) | 1905.50 | 0.33 |
| **Pulmonary valve (m/s)** | 1.20 (0.90, 1.50) | 1.00 (0.80, 1.30) | 1738.50 | 0.08 |
| **Degree of mitral valve regurgitation ( n, %)** |  |  | 3.93 | 0.27 |
| -None | 72 (86.75%) | 38 (74.51%) |  |  |
| -A small amount | 5 (6.02%) | 8 (15.69%) |  |  |
| -Medium | 4 (4.82%) | 3 (5.88%) |  |  |
| -A large amount | 2 (2.41%) | 2 (3.92%) |  |  |
| **Degree of tricuspid regurgitation** **( n, %)** |  |  | 11.63 | 0.009^*^ |
| -None | 10 (12.05%) | 4 (7.84%) |  |  |
| -A small amount | 36 (43.37%) | 13 (25.49%) |  |  |
| -Medium | 29 (34.94%) | 18 (35.29%) |  |  |
| -A large amount | 8 (9.64%) | 16 (31.37%) |  |  |
| **Degree of aortic valve regurgitation ( n, %)** |  |  | 1.51 | 0.68 |
| -None | 76 (91.57%) | 44 (86.27%) |  |  |
| -A small amount | 3 (3.61%) | 3 (5.88%) |  |  |
| -Medium | 1 (1.20%) | 2 (3.92%) |  |  |
| -A large amount | 3 (3.61%) | 2 (3.92%) |  |  |
| **Degree of pulmonary valve regurgitation ( n, %)** |  |  | 5.63 | 0.13 |
| -None | 65 (78.31%) | 36 (70.59%) |  |  |
| -A small amount | 16 (19.28%) | 9 (17.65%) |  |  |
| -Medium | 1 (1.20%) | 5 (9.80%) |  |  |
| -A large amount | 1 (1.20%) | 1 (1.96%) |  |  |

^*^The Pearson correlation coefficient showed that R=0.260, P (Approx. Sig.) = 0.002 < 0.05, indicating a mild linear positive correlation between adverse neonatal outcomes and the degree of tricuspid regurgitation.

**Table S18 Comparison of ECG results in pregnant women with PAH with and without adverse neonatal outcomes**

|  | Control group (n=83) | Adverse outcome group (n=51) | X^2^ | P-value |
| --- | --- | --- | --- | --- |
| ECG results ( n, %) |  |  |  |  |
| -Sinus tachycardia | 17 (20.48%) | 9 (17.65%) | 0.16 | 0.69 |
| -ST-T changes | 7 (8.43%) | 10 (19.61%) | 3.56 | 0.06 |
| -T wave changes | 10 (12.05%) | 5 (9.80%) | 0.16 | 0.69 |
| -Left ventricular high voltage | 2 (2.41%) | 3 (5.88%) | 1.06 | 0.37* |
| -Right ventricular high voltage | 2 (2.41%) | 3 (5.88%) | 1.06 | 0.37* |
| -Right axis deviation | 8 (9.64%) | 13 (15.49%) | 6.01 | 0.01 |
| -Left atrial hypertrophy | 8 (9.64%) | 13 (15.49%) | 6.01 | 0.01 |
| -Right atrial hypertrophy | 0 (0.00%) | 2 (3.92%) | 3.30 | 0.14* |
| -Right ventricular hypertrophy | 8 (9.64%) | 7 (13.73%) | 0.53 | 0.47 |
| -Clockwise rotation | 7 (8.43%) | 4 (7.84%) | 0.02 | 0.90 |
| -Atrial fibrillation | 1 (1.20%) | 0 (0.00%) | 0.62 | 1.00* |
| -Abnormal Q wave | 3 (3.61%) | 3 (5.88%) | 0.38 | 0.67* |
| -Ventricular premature beats | 3 (3.61%) | 3 (5.88%) | 0.38 | 0.67* |
| -Incomplete right bundle branch block | 20 (24.10%) | 16 (31.37%) | 0.85 | 0.36 |
| -Complete right bundle branch block | 6 (7.23%) | 3 (5.88%) | 0.09 | 0.76* |

*Fisher test

**Table S19 Comparison of blood biochemical in pregnant women with PAH with and without adverse neonatal outcomes**

|  | Control group (n=83) | Adverse outcome group (n=51) | t/U | P-value |
| --- | --- | --- | --- | --- |
| **SpO_2_（%）** | 98.00 (97.00, 99.00) | 97.00 (95.00, 98.00) | 1384.50 | 0.00 |
| **TBIL（umol/L）** | 9.92 ± 5.78 | 11.42 ± 6.82 | -1.36 | 0.18 |
| **ALB（g/L）** | 32.44 ± 3.84 | 31.17 ± 4.93 | 1.66 | 0.10 |
| **WBC（10^12^/L）** | 8.66 ± 2.28 | 9.00 ± 2.84 | -0.78 | 0.44 |
| **Hb（g/L）** | 107.82 ± 18.09 | 128.67 ± 19.40 | -0.26 | 0.80 |
| **Hct（%）** | 33.00 ± 5.09 | 33.16 ± 5.46 | -0.17 | 0.87 |
| **RBC（10^12^/L）** | 3.72 ± 0.40 | 3.75 ± 0.75 | -0.27 | 0.79 |
| **PLT（10^9^/L）** | 207.53 ± 62.75 | 184.80 ± 51.38 | 2.18 | 0.03 |
| **TT（s）** | 16.20 (15.20, 17.00) | 16.30 (15.50, 17.00) | 2087.00 | 0.89 |
| **FIB（g/L）** | 4.27 (3.93, 4.90) | 4.32 (3.70, 5.06) | 2023.00 | 0.67 |
| **APTT（s）** | 32.3 (30.5, 34.7) | 33.3 (31.5, 34.6) | 1893.50 | 0.31 |
| **INR** | 0.95 (0.90, 1.01) | 0.94 (0.92, 1.03) | 1927.00 | 0.39 |
| **PT（s）** | 12.3 (11.8, 13.3) | 12.5 (12.2, 13.2) | 1933.00 | 0.40 |
| **D-D（mg/L）** | 1.04 (0.71, 1.69) | 0.89 (0.58, 1.85) | 1942.00 | 0.43 |
| **BNP（pg/ml）** | 64.70 (27.40, 202.00) | 486.90 (110.20, 858.30) | 1054.50 | 0.00 |
| **CK（U/L）** | 53.00 (34.00, 85.00) | 41.00 (27.00, 56.00) | 1696.50 | 0.05 |
| **LDH（U/L）** | 205.48 ± 81.19 | 250.66 ± 111.80 | -2.51 | 0.01 |

SpO2, oxyhemoglobin saturation. TBIL, total bilirubin. ALB, albumin. WBC, white blood cell. Hb, hemoglobin. Hct, hematocrit. RBC, red blood cell. PLT, platelet count. TT, thrombin time. FIB, fibrinogen. APTT, activated partial thromboplastin time. INR, activated partial thromboplastin time. PT, prothrombin time. D-D, D-dimer. BNP, B-type natriuretic peptide. CK, Creatine Kinase. LDH, lactate dehydrogenase.

**Table S20 Comparison of pregnancy outcomes in pregnant women with PAH with and without adverse neonatal outcomes**

|  | Control group (n=83) | Adverse outcome group (n=51) | X^2^/U | P-value |
| --- | --- | --- | --- | --- |
| **Methods of terminating pregnancy ( n, %)** |  |  | 12.12 | 0.001 |
| -Vaginal delivery | 3 (3.61%) | 0 (0.00%) |  |  |
| -Cesarean section to remove the fetus | 1 (1.20%) | 8 (15.69%) |  |  |
| -Cesarean delivery | 79 (95.18%) | 43 (84.30%) |  |  |
| **ICU admission** **( n, %)** |  |  | 4.82 | 0.04 |
| -No | 58 (69.88%) | 26 (51.10%) |  |  |
| -Yes | 25 (30.12%) | 25 (49.02%) |  |  |
| **Blood loss (ml)** | 300.00 (200.00, 300.00) | 300.00 (200.00, 300.00) | 2011.00 | 0.61 |
| **Anesthesia methods ( n, %)** |  |  | 1.22 | 0.54 |
| -No anesthesia | 3 (3.61%) | 2 (3.92%) |  |  |
| -Intraspinal anesthesia | 58 (69.88%) | 31 (60.78%) |  |  |
| -General anesthesia | 22 (26.51%) | 18 (35.29%) |  |  |
